# High utilization efficiencies of alkylbenzokynones hybridized inside the pores of activated carbon for electrochemical capacitor electrodes†

**DOI:** 10.1039/d2ra06634c

**Published:** 2023-01-17

**Authors:** Hiroyuki Itoi, Miku Matsuura, Yuichiro Tanabe, Shoya Kondo, Takanori Usami, Yoshimi Ohzawa

**Affiliations:** a Department of Applied Chemistry, Aichi Institute of Technology Yachigusa 1247, Yakusa-cho Toyota 470-0392 Japan itoi-hiroyuki@aitech.ac.jp; b Graduate School of Chemical Sciences and Engineering, Hokkaido University Kita 13, Nishi 8, Kita-ku Sapporo 060-8628 Japan

## Abstract

Benzoquinone derivatives (BQDs) are hybridized inside activated carbon (AC) pores *via* gas-phase adsorption to prepare electrochemical capacitor materials. In this study, 2 mmol of BQDs are hybridized with 1 g of AC. The hybridization of alkylbenzoquinones (ABQs) with AC enhances the volumetric capacitances of the hybrids from 117 to 201 F cm^−3^ at 0.05 A g^−1^ and the capacitances are retained up to 73% at 10 A g^−1^. Meanwhile, the volumetric capacitances are increased to 163 F cm^−3^ at 0.05 A g^−1^ by the hybridization of halobenzoquinones (HBQs) and the capacitance retentions at 0.05 A g^−1^ are ∼62%, which are higher than that of AC (46%). The results of electrochemical measurements suggest that HBQs exist as agglomerates while ABQs are finely dispersed inside the pores. The ABQs have good contact with the conductive carbon pore surface compared to the HBQs. Consequently, most of the ABQ molecules undergo reversible redox reactions (*i.e.*, high utilization efficiencies), and a large contact area facilitates charge transfer at the large contact interface, thereby endowing the hybrids of ABQs with fast charging and discharging characteristics. HBQ molecules can be finely dispersed by liquid-phase adsorption, but the finely dispersed HBQ molecules are mobile inside the pores at room temperature and gradually form agglomerates. The difference in the existing form of BQDs is explained by the dominant interaction affecting the BQD molecules. ABQs have a strong interaction with the carbon pore surface while the intermolecular interaction is dominant for HBQs.

## Abstract 2

Benzoquinone derivatives are hybridized inside the pores of activated carbon *via* gas-phase adsorption. Alkylbenzoquinones have strong interaction with the carbon pore surface while the intermolecular interaction is dominant for halobenzoquinones.

## Graphical abstract

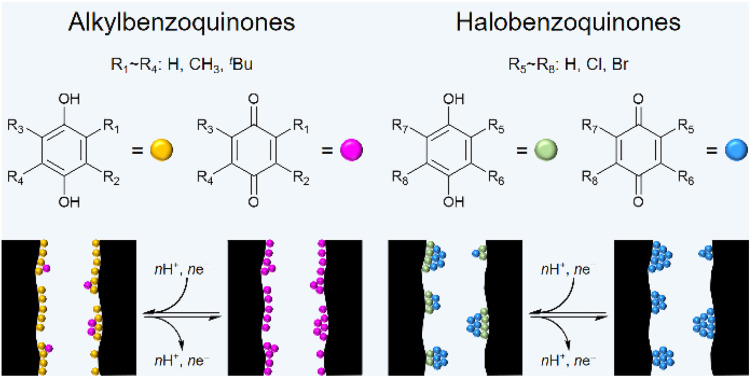

## 1. Introduction

The energy densities of electrochemical capacitor materials can be enhanced by the hybridization of redox-active materials with conductive substrates.^1–3^ A large contact interface is required for the hybrids toward high-performance electrochemical capacitor materials.^4^ Because many redox-active materials such as redox-active organic compounds^5,6^ and metal oxides^7,8^ have poor electrical conductivities despite their high energy densities^9,10^ and all the hybridized redox-active materials do not necessarily take part in the redox-reactions, especially at high current densities. A large contact interface facilitates charge transfer at the contact interface for balancing high power and high energy densities and increases the utilization efficiencies of the hybridized redox-active materials.^11^ Most hybridization techniques, however, require multi-step processes and organic solvents, and all of the redox-active materials cannot be completely hybridized with conductive substrates without any loss. Therefore, the hybridization must be performed in reduced steps in terms of maximizing the resource use.

We have recently reported the hybridization of redox-active materials, such as conductive polymers,^12,13^ metal oxides,^14^ organometallic complexes,^15,16^ and redox-active organic compounds,^17–20^ inside porous carbon pores to obtain high-performance electrode materials. Since porous carbons have high surface areas, large pore volumes, and high electrical conductivities, a large amount of redox-active materials can be introduced inside the pores with large contact areas between the hybridized redox-active materials and conductive carbon surfaces, thereby enabling rapid transfer of a large amount of charge at the large contact interface.^21^ Moreover, the introduction of redox-active materials does not increase the volume of porous carbon particles, and strong adsorption capabilities of porous carbons suppress desorption of redox-active materials. Consequently, high volumetric energy densities and long cycle lifetimes can be balanced with high power densities, which is very important for the practical applications of electrical energy storage devices. However, we regard the utilization efficiencies of hybridized redox-active materials as another important parameter for discussing electrode performances.

By using a gas-phase adsorption technique, redox-active organic compounds are completely hybridized inside porous carbon pores in a single-step process unless the amounts of redox-active materials exceed the saturation amounts of porous carbons.^17–20^ This hybridization method can accurately control the weight ratios of redox-active organic compounds and porous carbons, and therefore enables a quantitative discussion on the utilization efficiencies of the hybridized redox-active organic compounds. We have studied the electrochemical capacitor performances of the hybrids prepared from porous carbons and a benzoquinone derivative (BQD), 2,5-dichloro-1,4-benzoquinone (DCBQ), *via* gas-phase adsorption.^17,19,20^ BQDs have an advantage of the two-electron redox reaction over the redox-active materials involved by one-electron redox reaction for enhancing the energy densities of electrode materials. Proton conduction plays an important role in the redox reactions of BQDs in proton acidic electrolytes and is based on the Grotthuss mechanism inside porous carbon pores.^19^ By using aqueous H_2_SO_4_ electrolyte, the volumetric capacitance of the hybrids is enhanced by several times compared to those of pristine porous carbons. Moreover, the hybrids show superior capacitance retentions at high current densities over those of porous carbons, which are used as electrodes for electric-double layer capacitors. Accordingly, the power densities of the hybrids are not balanced with those of battery electrodes, and therefore, the hybrids are used as electrochemical capacitor electrodes. This hybridization does not need to fix BQDs onto conductive carbon surfaces through immobilization techniques as far as BQDs have enough hydrophobicity to suppress desorption in aqueous H_2_SO_4_ electrolyte.^22^ However, not all the BQDs molecules contribute to the capacitance enhancement despite the large contact interface. The utilization efficiencies of DCBQ and the power densities of the hybrids gradually decrease with the increasing amount of DCBQ due to the disturbance of proton conduction inside the DCBQ-constrained pores.^17,20^ Meanwhile, the proton conduction is facilitated by mesopores, whereas the utilization efficiencies decrease with increasing mesopore sizes.^19,20^ The utilization efficiency of DCBQ also depends on the pore structure and pore sizes of porous carbons^19^ and is maximized to *ca.* 50% by the mesopores of ∼4 nm and appropriately remaining mesopore volume despite its high volumetric capacitance enhancement. Therefore, it is of great importance to reveal what affects and how to increase the utilization efficiencies of BQDs for further enhancing the energy density of electrochemical capacitors.

In this study, 2 mmol of BQDs are hybridized with 1 g of activated carbon (AC) through gas-phase adsorption to quantitatively study the effects of their molecular structures on the utilization efficiencies of BQDs and the charging and discharging characteristics of the hybrids. It is found that there are large differences in the utilization efficiency and power density between the hybrids of halobenzoquinones (HBQs) and those of alkylbenzoquinones (ABQs). The differences are probably ascribed to the existing form of the hybridized BQDs inside the AC pores.

## 2. Experimental section

### 2.1. Materials and synthesis

Activated carbon (AC; MSC30, Kansai Coke and Chemicals Co., Ltd), 2,6-dimethyl-1,4-benzoquinone (*m*-DMBQ, 99%), tetramethyl-1,4-benzoquinone (TMBQ, 97%), *tert*-butyl-1,4-benzoquinone (^*t*^BuBQ, >98.0%), 2,6-di-*tert*-butyl-1,4-benzoquinone (*m*-D^*t*^BuBQ, 98%), 1,4-benzoquinone (BQ, >98.0%), 2,5-dichloro-1,4-benzoquinone (*p*-DCBQ, 98%), 2,6-dichloro-1,4-benzoquinone (*m*-DCBQ, >98.0%), tetrachloro-1,4-benzoquinone (TCBQ, 99%), and 2,5-dibromo-1,4-benzoquinone (*p*-DBrBQ, >98.0%) were used without any pretreatment or purification. BQ was purchased from FUJIFILM Wako Pure Chemical Corp., *m*-DMBQ, TMBQ, *m*-D^*t*^BuBQ, *p*-DCBQ, and TCBQ were purchaced from Sigma-Aldrich, and the other BQDs were purchased from Tokyo Chemical Industry Co., Ltd. The hybrids of AC and the BQDs were synthesized as described previously.^20^ Briefly, 1 g of the previously dried AC was mixed with 2 mmol of BQD in a glass vial and the glass vial was sealed under nitrogen atmosphere, followed by keeping the glass vial at a constant temperature for 24 h (Table S4†). The liquid-phase adsorption was performed by mixing AC and *p*-DCBQ in dichloromethane at 25 °C for 24 h. The solvent was evaporated and completely removed under vacuum at room temperature for 2 h.

### 2.2. Characterization

X-ray diffraction (XRD), nitrogen adsorption/desorption, and Raman spectroscopy analyses were performed according to the methods described previously.^19,20^ The Brunauer–Emmett–Teller (BET) specific surface area was calculated using the data of the adsorption isotherm in a *P*/*P*_0_ range of 0.05 to 0.20. Total pore volumes were determined from the adsorbed amount of nitrogen at *P*/*P*_0_ of 0.96. The Dubinin–Radushkevich method was applied to estimate micropore volumes. Mesopore volumes were calculated by subtracting micropore volumes from total pore volumes. Pore size distributions were calculated by the density functional theory (DFT) method based on the carbon slit pores (77 K, N_2_).

### 2.3. Electrochemical measurement

Electrochemical measurements were conducted at 25 °C using three-electrode and two-electrode cells. Aqueous 1 M H_2_SO_4_ electrolyte and a Ag/AgCl reference electrode were used to construct the cells. The detailed method of the electrode preparation is explained in the ESI† and the apparatuses for the electrochemical measurements are described in our previous works.^19^ Before measurements, electrodes were immersed in the electrolyte solution under vacuum for 1 h with the exception of the hybrid of AC and BQ (*i.e.*, AC/BQ). In the case of AC/BQ, the immersion time was shortened to 10 min to prevent the desorption of BQ. In three-electrode cell measurements, cyclic voltammetry (CV) was done at 1 mV s^−1^ between −0.1 and 0.8 V (*vs*. Ag/AgCl). After the CV, an impedance spectrum was collected at anodic peak top potentials of the BQDs, followed by galvanostatic charge/discharge measurement (GC). CV was again performed to confirm the degradation of the working electrode. Finally, the CV of the counter electrode was carried out to confirm the elution of BQDs from the working electrode and their subsequent adsorption on the counter electrode. The volumetric current in the voltammograms was calculated from the following equation (for details, see the ESI†):^16,20,23,24^1
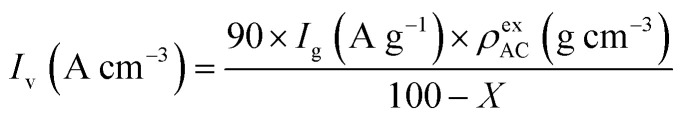
where *I*_g_ is the gravimetric current (*i.e.*, the current per 1 g of AC or AC/BQD hybrids), *ρ*^ex^_AC_ is the electrode density of AC (*i.e.*, 0.33 g cm^−3^),^19^*X* is the weight percentage of BQD in the AC/BQD hybrids. The gravimetric capacitance (*C*_g_ F g^−1^) was calculated according to the following equation:2
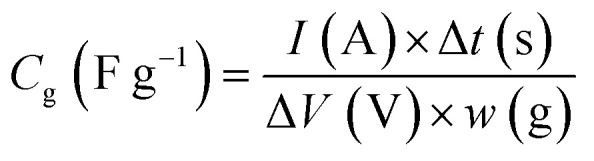
where *I* is the current, Δ*t* is the time from −0.1 to 0.8 V, Δ*V* is the potential window (*i.e.*, 0.9 V) despite the current density, and *w* is the weight of AC or AC/BQD hybrids. As well as the volumetric current, the volumetric capacitance (*C*_v_ F cm^−3^) was calculated using the following equation:3
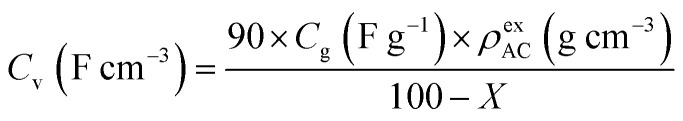


In two-electrode cell measurements, the weights of the anode and cathode were determined by balancing the gravimetric capacitance at 2 A g^−1^ measured using a three-electrode cell (Table S2†). The cell voltage was between 0 and 0.8 V and the sweep rate of CV was 1.0 mV s^−1^. The current density of the cycle test was 1.0 A g^−1^. The volumetric current and volumetric capacitance for the symmetrical cell of AC were calculated using eqn (1) and (3), respectively (*i.e.*, *X* = 0). For the asymmetrical two-electrode cell prepared using AC and the hybrid of AC and TMBQ (*i.e.*, AC/TMBQ), the volumetric current and volumetric capacitance were calculated according to the following equations:4
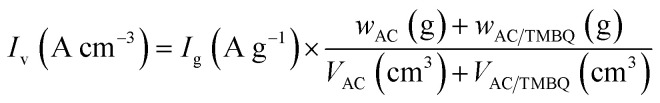
5
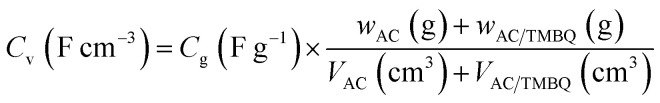
where *w*_AC_ and *V*_AC_ are the weight and volume of the electrode of AC (cathode), respectively; *w*_AC/TMBQ_ and *V*_AC/TMBQ_ are the weight and volume of the electrode of AC/TMBQ (anode), respectively. The gravimetric energy density (*E*_g_ W h kg^−1^) and gravimetric power density (*P*_g_ W kg^−1^) were calculated using the following equations:^25,26^6
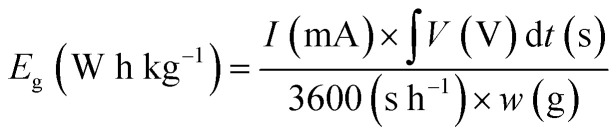
7
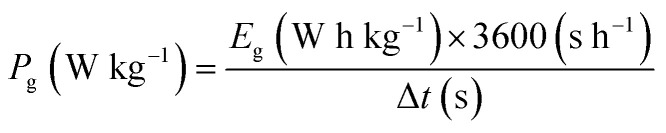
where Δ*t* is the time from 0.8 to 0 V. The volumetric energy density (*E*_V_ W h L^−1^) of the symmetrical cell was calculated based on eqn (1) (*i.e.*, *X* = 0), according to the following equation:

8 *E*_V_ (W h L^−1^) = 0.9 × *E*_g_ (W h kg^−1^) × *ρ*^ex^_AC_ (g cm^−3^)

The volumetric energy density of the asymmetrical cell was calculated based on eqn (4) and (5) using the following equation:9
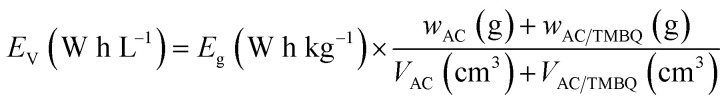


The volumetric power density (*P*_v_ W L^−1^) was calculated using the following equations:10
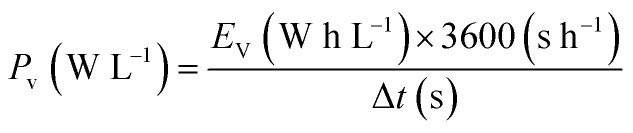


## 3. Results and discussion

### 3.1. Structural characterization

**Fig. 1:**
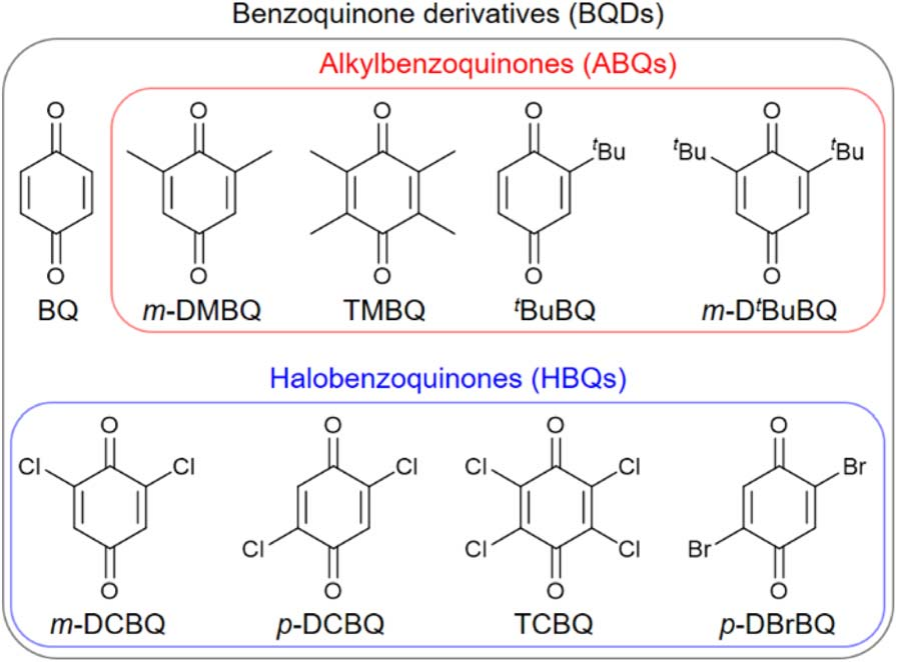
Structures of the BQDs examined in this study.

The benzoquinone derivatives (BQDs) shown in Fig. 1 were hybridized with KOH-activated carbon (AC) with a BET surface area of 3160 m^2^ g^−1^ and a total pore volume of 1.59 cm^3^ g^−1^. The pores of AC consist of micropores and mesopores of ∼4 nm,^27^ and the mesopores of ∼4 nm has been revealed to facilitate proton conduction inside the AC pores, which is based on the Grotthuss mechanism.^19^ As shown in Fig. 1, we examined four kinds of alkylbenzoquinones (ABQs): 2,6-dimethyl-1,4-benzoquinone (*m*-DMBQ), tetramethyl-1,4-benzoquinone (TMBQ), *tert*-butyl-1,4-benzoquinone (^*t*^BuBQ), and 2,6-di-*tert*-butyl-1,4-benzoquinone (*m*-D^*t*^BuBQ). For comparison with the ABQs, four kinds of halobenzoquinones (HBQs) were examined: 2,6-dichloro-1,4-benzoquinone (*m*-DCBQ), 2,5-dichloro-1,4-benzoquinone (*p*-DCBQ), tetrachloro-1,4-benzoquinone (TCBQ), and 2,5-dibromo-1,4-benzoquinone (*p*-DBrBQ). In addition, 1,4-benzoquinone (BQ) was used as an intermediate between the ABQs and HBQs. BQDs were adsorbed on AC through gas-phase adsorption, whereby the dried AC was directly mixed with BQD and the mixture was subsequently heated in a glass vial for 24 h. The amounts of the BQDs were fixed to 2 mmol per 1 g of the dried AC, which are much smaller than the saturation amounts of AC (*vide infra*), to quantitatively discuss the effects of their molecular structures on the electrochemical capacitor performance. The resulting hybrids are denoted as AC/Y, where Y represents the abbreviation of the hybridized BQD.

Adsorption of the BQDs inside the AC pores can be confirmed by X-ray diffraction (XRD) analysis, nitrogen adsorption/desorption measurement, and scanning electron microscopy (SEM) observation (Fig. S4–S8†). The XRD patterns of all hybrids did not show any distinct peaks like bulk BQDs but showed a broad peak, indicating that there was little BQD molecules on the surface of AC particles (Fig. S4†). The results of the nitrogen adsorption/desorption measurements showed that the adsorbed BQDs did not localize in micropores or mesopores but were distributed in both micropores and mesopores (Fig. S5 and Table S5†). This is in good agreement with the results of the hybrids prepared from porous carbons and a variety of organic and organometallic molecules *via* gas-phase adsorption.^13,17–20^ Since there remains a large amount of micropore and mesopore volumes for all hybrids, the BQD molecules do not disturb the proton conduction inside the AC pores, which is necessary for the redox reactions of BQDs.^19,20^ The SEM observation did not identify any difference in the morphology between AC and the AC/BQD hybrids (Fig. S6–S8†). The Raman spectroscopy analysis of the hybrids revealed that there was little difference in the ratios of the D and G band intensities (Fig. S9†), indicating that the hybridization was not accompanied by the pore collapse of AC or charge transfer between AC and the BQDs.^19^

### 3.2. Electrochemical behavior

**Fig. 2:**
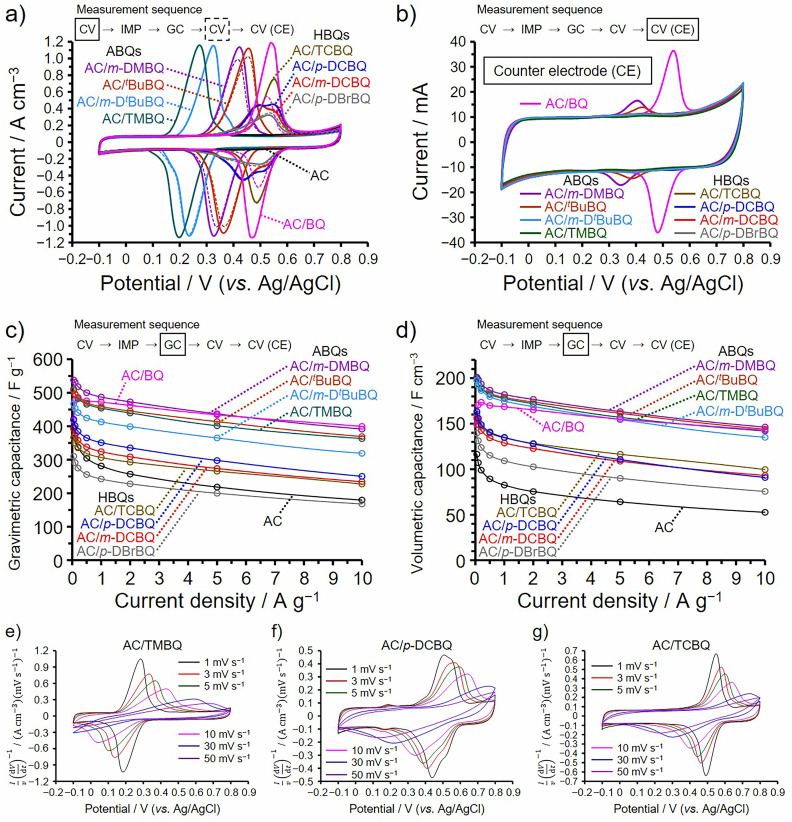
Results of electrochemical measurements performed at 25 °C using aqueous 1 M H_2_SO_4_ electrolyte and a three-electrode cell. (a) Cyclic voltammograms collected at 1 mV s^−1^ between −0.1 and 0.8 V. Solid and dashed lines correspond to the voltammograms before impedance (Fig. 3) and after GC (Fig. 2c and d) analyses, respectively. (b) Cyclic voltammograms of the counter electrodes collected at 1 mV s^−1^ between −0.1 and 0.8 V after all electrochemical measurements. (c) Gravimetric and (d) volumetric capacitances measured by the GC analysis. (e–g) Cyclic voltammograms of (e) AC/TMBQ, (f) AC/*p*-DCBQ, and (g) AC/TCBQ collected at different scan rates.

All electrochemical measurements in Fig. 2 were performed using a three-electrode cell and aqueous 1 M H_2_SO_4_ electrolyte in a potential range from −0.1 to 0.8 V (*vs.* Ag/AgCl). Before measurements, the working electrodes of the AC/ABQ and AC/HBQ hybrids, which are the hybrids of AC with ABQs and HBQs, respectively, were immersed in the electrolyte solution under vacuum for 1 h. We had confirmed that 1 h is enough to fully fill the pores with the electrolyte solution. Meanwhile, since BQ is readily desorbed from AC in the electrolyte solution (*vide infra*), the immersion time was shortened to 10 min for AC/BQ. Cyclic voltammetry (CV) was first conducted at 1 mV s^−1^ and an impedance spectrum was then collected at the anodic peak top potentials of the BQDs, followed by galvanostatic charge/discharge (GC) analysis. In order to confirm the desorption of the BQDs during the electrochemical measurements, CV was again conducted and finally the counter electrode was analyzed by CV. The measurement sequence and the corresponding measurement are indicated in each figure (Fig. 2). As shown in Fig. 2a, the voltammograms collected before the impedance analysis and after the GC analysis are shown as solid and dashed lines, respectively. AC shows a typical rectangular-shaped voltammogram and the constant current is derived from the electric double-layer (EDL) formation, representing typical electric double-layer capacitor (EDLC) behavior. Note that the voltammograms in Fig. 2a are expressed as the current per unit electrode volume (*i.e.*, volumetric current). Because the hybridization does not expand the volume of the AC particles, and the amount of AC per unit electrode volume remains constant for all the AC/BQD hybrids (Fig. S2†).^13,15–20^ Indeed, all the hybrids show almost the same EDL-derived current between −0.1 and 0.1 V as that of AC, indicating that EDL formation was not disturbed by 2 mmol of the BQDs per 1 g of AC at 1 mV s^−1^. All the hybrids exhibit reversible peak couples due to the reversible redox reactions of the BQDs inside the AC pores. The redox potentials of the AC/ABQ hybrids decrease with the increasing electron-donating effect of alkyl groups compared to that of AC/BQ.^28^ The voltammograms of AC/TMBQ and AC/*m*-D^*t*^BuBQ collected after the GC analysis do not show a decrease in the peak current, indicating that TMBQ and *m*-D^*t*^BuBQ have enough hydrophobicity to suppress the desorption during the electrochemical measurements. However, AC/BQ, AC/*m*-DMBQ, and AC/^*t*^BuBQ show a decrease in the peak current after the GC analysis due to the desorption, suggesting the low hydrophobicities of BQ, *m*-DMBQ, and ^*t*^BuBQ. A significant decrease in the peak current was observed for AC/BQ because BQ does not have any hydrophobic groups. As shown in Fig. 2b, the desorption of BQDs can be also confirmed from the cyclic voltammograms of the counter electrodes. Since the weight of the counter electrode was the same for each cell, the counter electrodes of the AC/BQD hybrids show the same EDL-derived current. The peak current of the voltammogram in Fig. 2b increases with increasing the difference in the peak current between the voltammograms collected before the impedance analysis and after the GC analysis (Fig. 2a), indicating that the desorbed BQD molecules in the electrolyte solution were adsorbed on the counter electrode. On the other hand, the AC/HBQ hybrids do not show any decrease in the peak current after the GC analysis (Fig. 2a), and the counter electrodes do not show any peaks in their voltammograms (Fig. 2b). The high hydrophobicities of the HBQs are attributed to halogen atoms and the HBQs have enough hydrophobicities to suppress the desorption.

The present hybridization depends on physisorption, and the desorption should be avoided for the practical use of the hybrids as electrochemical capacitor materials. Desorption of hybridized redox-active materials is induced by the following two reasons. First, electrolyte solutions incur the desorption of the hybridized redox-active materials upon immersing the electrodes into electrolyte solutions, which can be readily suppressed by using hydrophobic redox-active materials for aqueous electrochemical capacitors.^17,19^ Second, redox-active materials are positively or negatively charged during charging, and the charged redox-active materials are pulled to the oppositely charged counter electrode.^15,27,29^ Protonation of BQDs in proton acidic electrolytes upon reduction plays an important role to retain the electrical neutrality of BQDs during charging, by forming hydroquinone derivatives. Moreover, proton conduction proceeds based on the Grotthuss mechanism in proton acidic electrolytes, and the protonation is facilitated by mesopores.^19^ Therefore, the fast protonation of BQDs within the AC pores suppressed the desorption. Desorbed hydroquinone derivatives are oxidized on the counterpart electrode when its potential increases enough to oxidize the desorbed hydroquinone derivatives, leading to a decrease in the potential of the counterpart electrode and a concomitant decrease in the cell voltage.^30^

Since the amounts of the BQDs are 2 mmol per 1 g of AC for all the hybrids, the quantity of electricity in the peak current should be constant as far as all the hybridized molecules take part in the redox reaction without desorption. However, AC/BQ and the AC/ABQ hybrids clearly show larger peak areas than those of the AC/HBQ hybrids because the utilization efficiencies of BQ and the ABQs are higher than those of the HBQs. Table 1 summarizes the utilization efficiencies of the BQDs in the hybrids calculated using the voltammograms in Fig. 2a (Fig. S10†). In order to exclude the effect of the desorption of the BQDs, the voltammograms collected before the impedance analysis were used for the calculation. The utilization efficiencies of BQ and the ABQs are as much as 84–91% while the HBQs show low utilization efficiencies of 32–49%. Clearly, halogen atoms decrease the utilization efficiency. The utilization efficiency indicates the ratio of the BQDs undergoing redox reactions on the conductive carbon pore surface and would be related to their existing form inside the AC pores (*vide infra*).

**Table 1:** Weight percentages, utilization efficiencies, capacitance retentions, bulk solution resistances, and charge transfer resistances of the AC/BQD hybrids

BQDs	*m*-DMBQ	TMBQ	^*t*^BuBQ	*m*-D^*t*^BuBQ	BQ	*m*-DCBQ	*p*-DCBQ	TCBQ	*p*-DBrBQ
Wt [%]	21.4	24.7	24.7	30.6	17.8	26.1	26.1	33.0	34.7
Utilization efficiency [%]^a^	89	87	91	91	84	45	49	49	32
Capacitance retention [%]^b^	73	71	73	68	82	60	56	62	54
Bulk solution resistance [Ω]^c^	0.23	0.17	0.22	0.28	0.22	0.19	0.15	0.19	0.16
Charge transfer resistance [Ω]^c^	0.42	0.25	0.41	0.38	0.20	0.40	0.35	0.33	0.44

a Utilization efficiencies were calculated from the anodic peak current of the voltammogram in Fig. 2a.

b Capacitance retentions at 10 A g^−1^ based on the maximum capacitance.

c The bulk solution resistance and charge transfer resistance of AC are 0.14 and 0.48, respectively.

Fig. 2c and d show the dependence of the gravimetric and volumetric capacitances on the current density, respectively, determined by the GC measurement (Fig. S11–S15†). Table 1 includes the capacitance retentions at 10 A g^−1^ compared to the maximum capacitances, which correspond to the capacitances at 0.05 A g^−1^ for the hybrids except for AC/BQ. Meanwhile, AC/BQ shows the lower gravimetric and volumetric capacitances at 0.05 and 0.1 A g^−1^ than those at 0.2 A g^−1^. Note that the capacitance retentions calculated using the gravimetric capacitances are the same as those calculated using the volumetric one, and the GC analysis was conducted from 10 to 0.05 A g^−1^ to minimize the effect of the desorption of BQDs, especially at time-consuming current densities from 0.05 to 0.1 A g^−1^. Since BQ in AC/BQ remains the reduced form (*i.e.*, hydroquinone) between −0.1 and 0.3 V (Fig. 2a), the resulting hydrophilic hydroquinone was readily desorbed from the AC pores at such time-consuming current densities, which is confirmed from the voltammogram collected after the GC analysis (Fig. 2a) and that of the counter electrode (Fig. 2b). The dependence of the enhancements in the gravimetric capacitance is obviously different from that in the volumetric one. The gravimetric capacitance is correlated with not only the utilization efficiencies of the BQDs but also the weight percentages of the BQDs in the hybrids (Table 1). Even if all the BQDs show the same utilization efficiencies, the hybrids of the BQDs with large molecular weights show small enhancements in the gravimetric capacitance. On the other hand, the volumetric capacitance is simply related to the utilization efficiencies of the BQDs and are not correlated with the molecular weights of the BQDs. Because the present hybridization does not expand the AC particles and therefore enhances the volumetric capacitance of the hybrids.^13,15–20^ The enhancement in the volumetric capacitance is higher than that in the gravimetric one and this is advantageous for the development of practical electrode materials. The AC/ABQ hybrids and AC/BQ show high volumetric capacitances because their utilization efficiencies are as much as ≥84%. The volumetric capacitances of the AC/ABQs are at least 2.0 times higher than those of AC over 0.2 A g^−1^, which is a practical current density region for electrochemical capacitors. On the other hand, the AC/HBQ hybrids show low volumetric capacitance enhancements due to the low utilization efficiencies of the HBQs. Nevertheless, the volumetric capacitances of the AC/HBQs are higher than those of AC over the whole current density region. The capacitance retention of AC is as much as 46% because the mesopores of AC facilitate ion diffusion within the AC pores.^31,32^ Notably, the capacitance retentions of the AC/ABQ hybrids and AC/BQ are much higher than that of AC, despite the high utilization efficiencies and concomitantly substantial charge transfer of ABQs and BQ. This result indicates that the charge transfer at a large contact interface proceeds more rapidly than the EDL formation.^12,13,16–20^ Meanwhile, the AC/HBQ hybrids show lower capacitance retentions than those of the AC/ABQ hybrids and AC/BQ, whereas their capacitance retentions are still higher than that of AC. Fig. 2e–g show the voltammograms of AC/TMBQ, AC/*p*-DCBQ, and AC/TCBQ, which were collected at different sweep rates. For a clear comparison between the voltammograms collected at high and low sweep rates, the current in each voltammogram was divided by the sweep rate. The selection of these hybrids is appropriate for comparison because AC/TMBQ shows the lowest peak potential while the peak potentials of AC/*p*-DCBQ and AC/TCBQ are highest among the AC/BQD hybrids. In addition, they do not show a decrease in the peak current due to enough hydrophobicity. All the voltammograms are characteristics of the redox-active behavior at high sweep rates despite the kind of BQDs, suggesting that the BQDs undergo reversible redox reactions at high charging and discharging rates.^33^ This is supported by the GC curves of the AC/BQD hybrids at high current densities (Fig. S11–S15†). The capacitance retention of AC/BQ is more or less overestimated because only the capacitance retention of AC/BQ was calculated by comparing with the capacitance at 0.2 A g^−1^ owing to the desorption of BQ below 0.2 A g^−1^. Due to the same reason, the utilization efficiency of AC/BQ would be slightly underestimated, whereas the time for the electrolyte immersion of AC/BQ was shortened to suppress the desorption of BQ. The number of halogen atom, dipole moment, and the degree of an electron withdrawing effect do not affect the utilization efficiencies and capacitance retentions of the AC/HBQ hybrids, considering the molecular structures of the HBQs. Similarly, the size of alkyl groups, dipole moment, or the degree of electron donating effect do not affect the utilization efficiencies and capacitance retentions of the AC/ABQ hybrids. This was further confirmed by using the hybrids prepared using the isomers of *m*-DMBQ and *m*-D^*t*^BuBQ (*i.e.*, AC/*p*-DMBQ and AC/*p*-D^*t*^BuBQ, Fig. S18†), and their volumetric capacitances and capacitance retentions do not differ depending on the kind or the position of the alkyl groups.

**Fig. 3:**
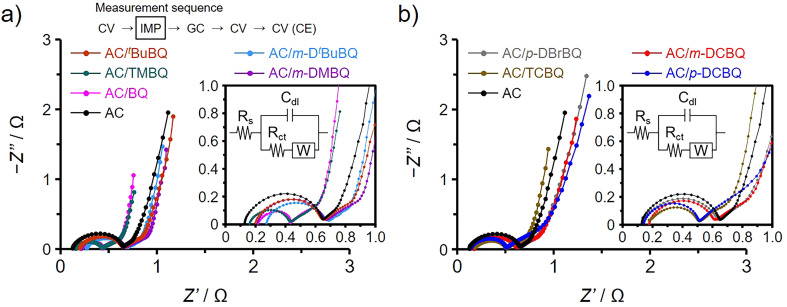
Nyquist plots collected at the anodic peak top potentials for (a) the AC/ABQ hybrids, AC/BQ, and AC and (b) the AC/HBQ hybrids and AC. Inset: enlarged Nyquist plots in the high frequency region and the equivalent circuit.

Fig. 3a and b show the Nyquist plots obtained by impedance analysis of the AC/ABQ and AC/HBQ hybrids, respectively. The charge transfer resistance increases with the size of the semicircle in the spectrum collected at high frequencies,^34–36^ while the ion diffusion resistance has a correlation with the locus of the spectrum collected at low frequencies.^37^ There are the other resistance components affecting the performance of the electrochemical capacitor electrode: the bulk solution resistance and the interfacial resistances between the AC particles and between the current collector and the AC particles.^38–40^ However, the differences in these resistance components can be ignored because all the electrodes were prepared under the precisely controlled conditions (for details, see the ESI†).^35^ The fitting of the Nyquist plots was performed (Fig. 3, inset),^41^ and the results are summarized in Table 1. Indeed, the bulk solution resistances range from 0.14 to 0.28 Ω with a slight difference. Since the difference in the capacitance retention between AC/TMBQ and AC/^*t*^BuBQ is only 2% despite a relatively large difference between their charge transfer resistances among the AC/BQD hybrids, the differences in the locus at low frequencies and the size of the semicircles can be ignored for all the hybrids. As for AC/*m*-D^*t*^BuBQ, 36% of the pore volume of AC is occupied by the adsorbed *m*-D^*t*^BuBQ (Table S5†), which is the largest molecule among the BQDs used in this study. However, such a remaining pore volume is enough for the proton conduction inside the pores, which proceeds based on the Grotthuss mechanism.^19,20^ Therefore, their molecular sizes do not affect the diffusion resistance due to the small contents of the BQDs in the hybrids. Similarly, the occupied volume of AC is less than 25% for the AC/HBQ hybrids and a difference in the diffusion resistance must be negligible for the AC/HBQ hybrids. The semicircle of AC is ascribed to the charge transfer of oxygen-containing functional groups, whereas their capacitance enhancement effect is slight compared to those of the AC/BQD hybrids.^35,36^ Although the volumetric capacitance is significantly increased by the substantial charge transfer at the contact interface between the BQDs and the carbon pore surface, all the hybrids do not show an increase in the size of the semicircle compared to that of AC, indicating that the charge transfer resistances of all the hybrids are lower than that of AC. This is supported by the fitting results of the charge transfer resistances (Table 1); the charge transfer resistances of the AC/BQD hybrids (0.20–0.44 Ω) are lower than that of AC (0.48 Ω). These results reason that the charge transfer of oxygen-containing functional groups is substituted by that of the BQDs due to the reduced charge transfer resistance by the large contact interface.^13,16^ Although the capacitance retentions of the AC/ABQ hybrids are higher than those of the AC/HBQ hybrids, the difference in the capacitance retention is not explained by the results of the impedance analysis.

### 3.3. Existing forms of BQDs inside the AC pores

**Fig. 4:**
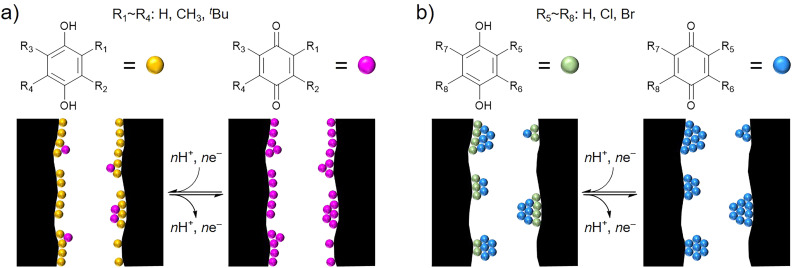
Plausible hybridization models dominated by the (a) interaction with the pore surface and (b) intermolecular interaction.

The utilization efficiencies of the ABQs are not affected by the size of the alkyl groups, the degree of the electron donating effect, or the dipole moment of the ABQs. Similarly, the utilization efficiencies of the HBQs are not affected by the degree of the electron withdrawing effect or the dipole moment of the HBQs. The utilization efficiencies and the capacitance retentions of the AC/BQD hybrids would be related to the contact between the BQDs and the conductive carbon pore surface, as shown in Fig. 4. Probably, most of the ABQ and BQ molecules are well dispersed inside the AC pores and have contact with the pore surface (Fig. 4a). Good contact of the ABQ and BQ molecules with the pore surface increases the utilization efficiency and facilitates charge transfer for enhancing the capacitance retention. Meanwhile, the low utilization efficiencies and low capacitance retentions of the HBQs are attributed to poor contact of the HBQ molecules with the pore surface, which is explained by the formation of agglomerates consisting of multiple HBQ molecules inside the AC pores (Fig. 4b). The HBQ molecules away from the pore surface do not undergo redox reactions especially at high current densities, thereby decreasing the utilization efficiency and capacitance retention. The existing form of the BQD molecules would be dominated by the interaction affecting the molecules: *i.e.*, the interaction with the pore surface and the intermolecular interaction. The dominant interaction on the ABQs and BQ is the one between the pore surface and molecules, while the agglomerate formation of the HBQs is dominated by the intermolecular interaction.

The interactions with the surfaces of graphene^42–47^ and graphene oxide^48–52^ have been reported based on the π–π stacking, hydrophobic, electrostatic, and hydrogen-bonding interactions.^53,54^ In the case of graphene oxide that has a variety of oxygen-containing functional groups, the electrostatic interaction is derived from the attracting force between positively and negatively charged functional groups,^49–52^ while the hydrogen bonding is induced between polar functional groups and/or polar molecular moieties.^50,53^ However, these electrostatic and hydrogen bonding interactions can be ignored on the pore surface of AC due to the small oxygen content of AC unlike graphene oxide. Similar to the interaction between the pore surface and molecules, the π–π stacking, hydrophobic, electrostatic, and hydrogen-bonding interactions must also affect the intermolecular interaction, but the electrostatic and hydrogen-bonding interactions can be ignored for the ABQs, BQ, and HBQs, considering the absence of polar or charged moieties in the BQD molecules.^55–59^ Therefore, the π–π stacking and hydrophobic interactions dominate the intermolecular interaction and the one between the pore surface and molecules. It cannot be determined whether the π–π stacking interaction or hydrophobic one dominates the intermolecular interaction or the one between the pore surface and molecules. Because not only halogen atoms but also many or bulky alkyl groups induce strong hydrophobic interaction, which certainly suppresses the elution of ABQs, as observed for AC/TMBQ and AC/*m*-D^*t*^BuBQ. Moreover, agglomerate formation or fine dispersion of HBQs depends on the way to hybridize AC and HBQs (*vide infra*).

**Fig. 5:**
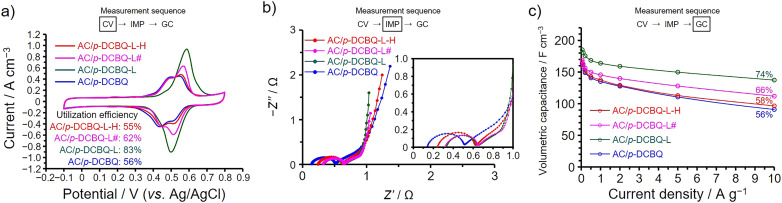
Results of electrochemical measurements conducted at 25 °C using aqueous 1 M H_2_SO_4_ electrolyte and a three-electrode cell for AC/*p*-DCBQ-L, AC/*p*-DCBQ-L-H, and AC/*p*-DCBQ-L#, shown alongside the results of AC/*p*-DCBQ for comparison. (a) Cyclic voltammograms collected at 1 mV s^−1^ between −0.1 and 0.8 V. (b) Nyquist plots collected at anodic peak top potentials. (c) Dependences of the volumetric capacitance on the current density measured by GC in a potential range from −0.1 to 0.8 V. The percentages in the figure are the capacitance retentions at 10 A g^−1^ based on the capacitances at 0.05 A g^−1^.

We then hybridized *p*-DCBQ with AC *via* the liquid-phase adsorption to examine whether the existing form of *p*-DCBQ inside the AC pores can be changed to enhance the utilization efficiency and capacitance retention. The amount of *p*-DCBQ in the hybrid is the same as that in AC/*p*-DCBQ and the hybrid is denoted as AC/*p*-DCBQ-L, where L represents to liquid-phase adsorption. In addition, we further conducted the heat treatment of AC/*p*-DCBQ-L under the same conditions as those for preparing AC/*p*-DCBQ in the gas phase (*i.e.*, 100 °C for 24 h). The resulting heat-treated hybrid is denoted as AC/*p*-DCBQ-L-H, where H represents to the heat treatment. Note that the heat treatment was not accompanied by desorption of *p*-DCBQ because a decrease in the weight of the hybrids was negligible after the heat treatment. In addition, there are no significant differences in the surface area (∼1.7%) and pore volume (∼1.4%) between AC/*p*-DCBQ, AC/*p*-DCBQ-L, and AC/*p*-DCBQ-L-H (Fig. S19 and Table S6†). Furthermore, AC/*p*-DCBQ-L had been left at room temperature for 5 months and the electrochemical measurements of this hybrid, denoted as AC/*p*-DCBQ-L#, was performed. The differences in the surface area and pore volume between AC/*p*-DCBQ-L and AC/*p*-DCBQ-L# are negligible (∼1%, Fig. S19 and Table S6†). Fig. 5 shows the results of electrochemical measurements for these hybrids, shown alongside the results of AC/*p*-DCBQ for comparison. Clearly, the peak area of the voltammogram of AC/*p*-DCBQ-L is increased compared to that for AC/*p*-DCBQ (Fig. 5a), and the utilization efficiency was increased to 83%. On the other hand, AC/*p*-DCBQ-L-H shows almost the same voltammogram as that of AC/*p*-DCBQ and the utilization efficiency is 55%, which is almost the same as that of AC/*p*-DCBQ (*i.e.*, 56%). Meanwhile, the shape of the voltammogram of AC/*p*-DCBQ-L# is intermediate between those of AC/*p*-DCBQ-L and AC/*p*-DCBQ-L-H and the utilization efficiency was 62%. The impedance spectra of AC/*p*-DCBQ-L, AC/*p*-DCBQ-L-H, and AC/*p*-DCBQ-L# show differences in the size of semicircle and the locus at low frequencies (Fig. 5b). The fitting results of the charge transfer resistances of AC/*p*-DCBQ-L, AC/*p*-DCBQ-L-H, and AC/*p*-DCBQ-L# were 0.29, 0.35, and 0.31 Ω, respectively. However, such differences were observed among the AC/ABQ hybrids that showed the high capacitance retentions and high utilization efficiencies and this is also the case with the AC/HBQ hybrids. By comparing with AC/*p*-DCBQ, the capacitance of AC/*p*-DCBQ-L was enhanced over the whole current density region and the capacitance retention based on the capacitance at 0.05 A g^−1^ is 74% (Fig. 5c). However, AC/*p*-DCBQ-L-H exhibits almost the same capacitance and capacitance retention (58%) as those of AC/*p*-DCBQ. The capacitance and capacitance retention of AC/*p*-DCBQ-L# are intermediate between those of AC/*p*-DCBQ-L and AC/*p*-DCBQ-L-H. From these results, it is obvious that the existing form of *p*-DCBQ in AC/*p*-DCBQ-L is different from those in AC/*p*-DCBQ and AC/*p*-DCBQ-L-H. Since the heat treatment of AC/*p*-DCBQ-L led to the decreases in the utilization efficiency and capacitance retention of AC/*p*-DCBQ-L-H, the heat treatment reduced the contact area between *p*-DCBQ and the pore surface, which can be explained by the agglomerate formation of *p*-DCBQ inside the pores upon the heat treatment. The agglomerate formation proceeds even at room temperature, as observed for AC/*p*-DCBQ-L#. This result suggests that *p*-DCBQ is mobile inside the AC pores at room temperature and the intermolecular interaction surpasses the interaction with the pore surface, whereas *p*-DCBQ is a solid at room temperature and has a melting point of *ca.* 160 °C under atmospheric pressure. The mobility of *p*-DCBQ inside the pores is enhanced by the heat treatment, accelerating the agglomerate formation of *p*-DCBQ. In the liquid phase, *p*-DCBQ was solvated and probably dispersed at the molecular level during the preparation of AC/*p*-DCBQ-L, and the solvated *p*-DCBQ was adsorbed inside the AC pores. Upon evaporation at room temperature, the solvating molecules were removed, leaving *p*-DCBQ molecules finely dispersed inside the AC pores. Meanwhile, *p*-DCBQ is agglomerated inside the AC pores in preparing AC/*p*-DCBQ in the gas phase due to the strong intermolecular interaction of *p*-DCBQ.

The advantage of the hybridization of AC and BQDs in the gas phase lies in the facility of the synthesis without any organic solvents or concomitant solvent removal and drying processes. In addition, the BQDs are completely adsorbed on porous carbons as far as the amounts of BQDs are less than the saturation amounts of porous carbons.^17,19,20^ Meanwhile, BQDs in the liquid phase cannot be completely adsorbed on porous carbons and solvent molecules are also adsorbed inside porous carbon pores through liquid-phase adsorption. Therefore, the maximum amounts of adsorbed BQDs in the liquid phase are much lower than the saturation amounts, which can be accomplished only in the gas phase. The present hybridization method achieves reproducibilities to accurately control the amount of adsorbed BQDs and quantitatively study the relation between the amounts of hybridized BQDs and electrochemical capacitor behaviors. Although the hybridization of HBQs with AC through the gas-phase adsorption enhances the volumetric capacitance and capacitance retention compared to those of AC, the utilization efficiencies and capacitance retentions of the hybrids are lower than those of the hybrids of ABQs due to the agglomerate formation of HBQs. The liquid-phase adsorption of *p*-DCBQ enhanced the electrochemical capacitor performance of AC/*p*-DCBQ-L, but the strong intermolecular interaction of *p*-DCBQ led to the agglomerate formation even at room temperature, resulting in the decrease in the electrochemical capacitor performance. On the other hand, the hybrids of ABQs prepared by gas-phase adsorption show the high utilization efficiencies and high capacitance retentions, and hydrophobicity necessary for suppressing desorption can be readily tuned by appropriate alkyl groups. Therefore, the use of ABQs are desirable for the practical preparation of high-performance electrochemical capacitor electrodes through the gas-phase adsorption.

### 3.4. Two-electrode cell measurements

**Fig. 6:**
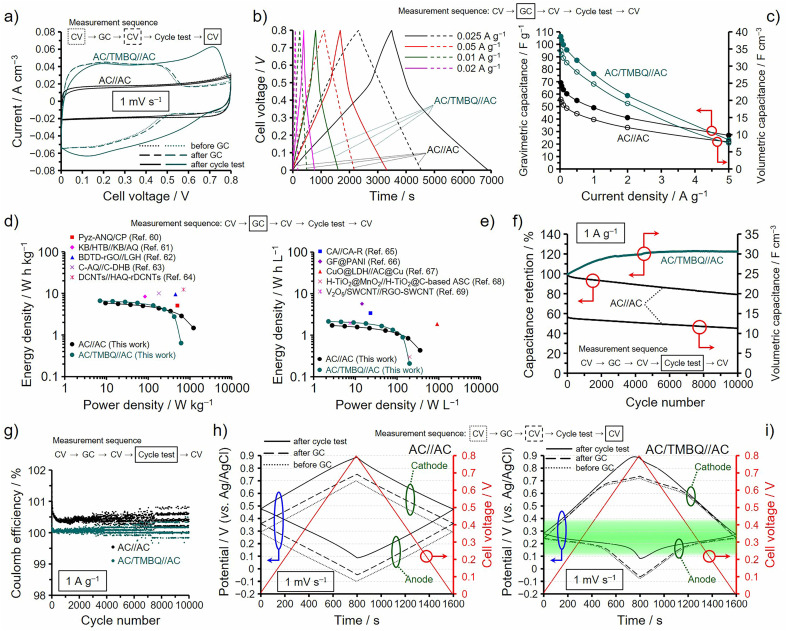
Results of electrochemical measurements for the symmetrical and asymmetrical two-electrode cells for AC//AC and AC/TMBQ//AC, respectively. The measurements were performed at 25 °C using aqueous 1 M H_2_SO_4_ electrolyte in a cell voltage from 0 to 0.8 V. (a) Cyclic voltammograms collected at 1 mV s^−1^. (b) GC curves collected by GC analysis. (c) Gravimetric and volumetric capacitances measured by GC analysis. (d and e) Ragone plots of (d) gravimetric energy and power densities and (e) volumetric energy and power densities, shown alongside reported values. (f) Results of cycle tests measured at 1 A g^−1^. (g) Dependences of the coulomb efficiencies on the cycle number. (h and i) Dependences of the cell voltage and the potentials of the anode and cathode measured during CV in (a) for the (h) symmetrical and (i) asymmetrical cells. The highlighted part in (i) indicates the redox potential range of TMBQ.

The hybrids of ABQs show high utilization efficiencies and high capacitance retentions, but hydrophobicity is important to suppress the desorption of ABQs for the practical use of the hybrids as electrochemical capacitor electrodes. We then performed electrochemical measurements by using a two-electrode asymmetrical cell consisting of AC/TMBQ and AC because TMBQ has enough hydrophobicity to suppress the desorption (Fig. 2a and b). AC/TMBQ was used as an anode due to its low redox potential while AC was used as a cathode. The weights of the anode and cathode were balanced considering the capacitances at 2 A g^−1^ determined by the GC analysis using a three-electrode cell (Table S2†). Fig. 6 shows the results of the two-electrode cell measurements of the asymmetrical cell, denoted as AC/TMBQ//AC. For comparison, the results for the symmetrical two-electrode cell of AC, denoted as AC//AC, are shown together. For both asymmetrical and symmetrical cell measurements, the cell voltage was regulated between 0 and 0.8 V while the potentials of the anodes and cathodes were measured using a reference electrode (for details, see the ESI†). In the measurements, CV was first conducted at 1 mV s^−1^ (Fig. 6a) and GC was then measured from 5 to 0.025 A g^−1^ (Fig. 6b–e), followed by CV (Fig. 6a). Subsequently, a cycle test was performed at 1 A g^−1^ for 10 000 cycles (Fig. 6f and g), and finally CV was measured again (Fig. 6a). As shown in Fig. 6a, the voltammograms of the symmetrical cell of AC show a slight decrease in the current after the GC and cycle test, but a significant difference between the voltammograms is not observed. Meanwhile, the asymmetrical cell shows a large increase in the current after the cycle test, whereas the voltammograms before and after the GC analysis do not show a large difference. The GC curves of the symmetrical cell are characterized by a linear dependence on the time while those of the asymmetrical cell show the change in the slope at *ca.* 0.5 V due to the occurrence of the redox reaction of TMBQ (Fig. 6b). The gravimetric and volumetric capacitances of the asymmetrical cell are more than 1.4 and 1.5 times higher, respectively, than those of AC up to 2 A g^−1^ (Fig. 6c), whereas the gravimetric and volumetric capacitances of the asymmetrical cell are comparable with those of the symmetrical cell at 5 A g^−1^. The gravimetric energy and power densities of the symmetrical and asymmetrical cells are plotted in Fig. 6d together with those of the reported electrochemical capacitors using redox-active organic compounds, such as Pyz-ANQ/CP (5.1 W h kg^−1^ at 0.5 kW kg^−1^),^60^ KB/HTB//KB/AQ (8.36 W h kg^−1^ at 83.6 W kg^−1^),^61^ BDTD-rGO//LGH (9.52 W h kg^−1^ at 0.45 kW kg^−1^),^62^ C-AQ//C-DHB (10 W h kg^−1^ at 0.18 kW kg^−1^),^63^ and DCNTs//HAQ-rDCNTs (12.3 W h kg^−1^ at 0.7 kW kg^−1^).^64^ In Fig. 6e, the volumetric energy and power densities of the symmetrical and asymmetrical cells are compared to those of the reported electrochemical capacitors using redox-active materials, such as CA//CA-R (3.4 W h L^−1^ at 23 W L^−1^),^65^ GF@PANI (5.7 W h L^−1^ at 14.5 W L^−1^),^66^ CuO@LDH//AC@Cu (1.857 W h L^−1^ at 914.5 W L^−1^),^67^ H-TiO_2_@MnO_2_//H-TiO_2_@C-based ASC (0.3 W h L^−1^ at 0.2 kW L^−1^),^68^ V_2_O_5_/SWCNT//RGO-SWCNT (1.95 W h L^−1^ at 7.5 W L^−1^).^69^ Both gravimetric and volumetric energy densities of AC/TMBQ//AC are comparable to or lower than those of the reported values at the corresponding gravimetric and volumetric power densities. AC was used as a cathode for the asymmetrical cell and its volume was 1.7 times larger than that of the anode for balancing the capacitances of the anode and cathode (Table S2†). Therefore, an increase in the amount of TMBQ in the anode and substituting other AC/BQD hybrids with appropriate redox potentials and optimized amounts of BQDs for the cathode can enhance the energy and power densities. The cycle test measured at 1 A g^−1^ revealed that the volumetric capacitance increased and slightly decreased for the asymmetrical and symmetrical cells, respectively (Fig. 6f). An increase in the volumetric capacitance after the cycle test was suggested from the enlarged voltammogram of the asymmetrical cell collected after the cycle test (Fig. 6a), and the volumetric capacitance of the asymmetrical cell is about 3 times higher than that of the symmetrical cell at the 10 000th cycle. The coulomb efficiencies of the asymmetrical and symmetrical cells shown in Fig. 6g suggest that the stored and released charges are well balanced for the asymmetrical cell compared to the symmetrical one. The long cycle lifetimes were also confirmed for the asymmetrical cells prepared using the hybrids of *p*-DCBQ with the same AC^19^ and other porous carbons with the average pore sizes of 5 to 30 nm.^20^Fig. 6h and i show the potential dependences of the anodes and cathodes during the CV in Fig. 6a for the symmetrical and asymmetrical cells, respectively, which were collected before and after the GC analysis and after the cycle test. For the symmetrical cell of AC (Fig. 6h), the initial potentials of the anode and cathode before the GC analysis are the same values of 0.29 V, where the cell voltage is 0 V. During the CV, the differences in their potentials are almost the half of the cell voltage (*i.e.*, *ca.* 0.4 V): −0.10 to 0.29 V for the anode and 0.29 to 0.70 V for the cathode. However, the potentials of the anode and cathode at the cell voltage of 0 V gradually increased after the GC and cycle test, which is probably attributed to the difference between the stored and released charges, as shown in Fig. 6g. Indeed, in the three-electrode cell measurements, AC showed higher irreversible anodic and cathodic currents than those of AC/TMBQ at ∼0.8 V and −0.1 V (*vs.* Ag/AgCl), respectively (Fig. S20†). Specifically, the difference in the irreversible anodic current is larger than that in the irreversible cathodic current between AC and AC/TMBQ (Fig. S20†), and therefore, the quantity of electricity to increase the cell voltage from 0 to 0.8 V is not equal to that required to decrease the cell voltage from 0.8 to 0 V for the symmetrical cell, which is the reason for its higher coulomb efficiency than 100%. The anodic current of AC is derived from oxidation of AC (Fig. S20†),^70^ suggesting that TMBQ suppresses the oxidation of AC. The suppression of oxidation was observed for the hybrids of *p*-DCBQ and zeolite-templated carbon,^19^ which is electrochemically oxidized much more easily than AC.^36^ On the other hand, the asymmetrical cell does not show a significant difference in the anodic and cathodic potentials at the cell voltage of 0 V after the GC analysis and shows a slight difference after the cycle test, which is suggested by almost the same stored and released charges: *i.e.*, an ideal coulomb efficiency. The potential windows of the anode for the asymmetrical cell after the GC and cycle test is between −0.06 and 0.25 V and 0.08 and 0.28 V, respectively (Fig. 6i). The difference in the potential window of the anode seems to be negligible, but the latter potential window is advantageous for the redox reaction of TMBQ in the anode. Because the potential range of the oxidation and reduction of TMBQ in AC/TMBQ is between 0.1–0.4 V (Fig. 2a), as highlighted in Fig. 6i. That is, the utilization efficiencies of TMBQ before and after the GC analysis are lower than that after the cycle test. Consequently, the potential window of the cathode after the cycle test was widened to 0.28–0.89 V for the asymmetrical cell by the increased utilization efficiency of TMBQ. In order not to decrease the utilization efficiencies of BQDs, not only the balance of the amounts of the anode and cathode but also the potential window of the redox reaction of BQDs must be considered. Otherwise, the utilization efficiencies of BQDs would decrease depending on the cell configuration. Fortunately, the redox potentials of ABQs are readily modified by appropriate alkyl groups with retaining sufficient hydrophobicity.

The hybrids of ABQs with high hydrophobicity balance the high utilization efficiency, high capacitance retention, and long cycle lifetimes. The amounts of the hybridized BQDs were 2 mmol per 1 g of AC in this study to compare the electrochemical capacitor performances of the AC/BQD hybrids, but there remains substantial pore volumes in the hybrids (Fig. S5 and Table S5†). Further introduction of ABQs into the AC pores will increase the volumetric capacitance with retaining the capacitance retention of the hybrids, but the introduction of a large amount of ABQs would disturb proton conduction, decreasing the utilization efficiencies of ABQs and capacitance retention.^17,19,20^ A further study is currently carried out to clarify the effects of the amount of ABQs on the electrochemical capacitor performances. The present hybridization through gas-phase adsorption is a one-step process and does not need any organic solvents or concomitant solvent removal. Moreover, as far as the amounts of BQDs do not exceed the saturation amounts of AC, all the BQD molecules are completely hybridized inside the AC pores.^17,19,20^ Furthermore, the redox potentials of ABQs depend on the structure of alkyl groups, and therefore, asymmetrical electrochemical capacitors are readily designed by changing the number and structure of alkyl groups. The present hybridization using ABQs with high hydrophobicities and appropriate redox potentials allows for a practical method to design high-performance electrochemical capacitors even using a small amount of ABQs due to their high utilization efficiencies.

## 4. Conclusion

In summary, we studied the effects of the molecular structures of the BQDs on the electrochemical capacitor performances of the AC/BQD hybrids. The ABQs and BQ showed the high utilization efficiencies and high capacitance retentions, but the ABQs with low hydrophobicity and BQ incurred the elution from the AC pores. Meanwhile, the HBQs showed the lower utilization efficiencies and capacitance retentions than those of the ABQs and BQ, whereas the HBQs showed high durability to the elution. The difference in the electrochemical capacitor performances is attributed to the existence form of the BQDs inside the AC pores. ABQs and BQ would be finely dispersed and have good contact with the conductive carbon pore surface, while HBQs are considered to form agglomerates consisting of multiple molecules. The existing form of *p*-DCBQ could be changed by liquid-phase adsorption, but the finely dispersed *p*-DCBQ molecules were gradually agglomerated at room temperature. This result suggests the mobile characteristics of *p*-DCBQ inside the AC pores, and the mobility was enhanced by the heat treatment. The hybrids of ABQs with high hydrophobicity can balance the high utilization efficiency, high capacitance retention, and long cycle lifetimes, thereby showing desirable electrochemical capacitor performance. A further study is currently conducted to clarify the effects of the amount of ABQs on the electrochemical capacitor performances. The present hybridization in the gas phase is a one-step process and does not need any organic solvents or concomitant solvent removal and drying processes. Moreover, all the BQD molecules are completely hybridized with AC as far as the amounts do not exceed the saturation amounts of AC. Furthermore, the utilization efficiencies of the ABQs were more than 87% and most of the hybridized ABQs contributed to the capacitance enhancement. Such high utilization efficiencies have not been obtained by any other synthetic method of the electrode materials in a single step. Therefore, the present hybridization allows for a practical method to prepare high-performance electrochemical capacitor electrodes in an environmentally friendly manner. Furthermore, the redox potentials of ABQs can be readily tuned by appropriate alkyl groups; therefore, high-performance asymmetrical electrochemical capacitors can be readily designed using AC/ABQ hybrids with appropriate redox potentials.

## Conflicts of interest

There are no conflicts to declare.

## Supplementary Material

RA-013-D2RA06634C-s001
