# Supplementary material for: High utilization efficiencies of alkylbenzokynones hybridized inside the pores of activated carbon for electrochemical capacitor electrodes
Source: RSC Adv. 2023 Jan 17;13(4):2587–99. doi: 10.1039/d2ra06634c (PMC9844457; doi:10.1039/d2ra06634c)
Supplement: RA-013-D2RA06634C-s001 [file RA-013-D2RA06634C-s001.pdf]

Electronic Supplementary Material (ESI) for RSC Advances.
This journal is © The Royal Society of Chemistry 2023

Supplementary Information

High Utilization Efficiencies of Alkylbenzokynones Hybridized inside the Pores of Activated Carbon for Electrochemical Capacitor Electrodes

Hiroyuki Itoi,[a] Miku Matsuura,[a] Yuichiro Tanabe,[a] Shoya Kondo,[b] Takanori Usami,[a] and Yoshimi Ohzawa[a]*

[a] Department of Applied Chemistry, Faculty of Engineering, Aichi Institute of Technology, 1247 Yachigusa, Yakusa, Toyota, 470−0392, Japan

[b] Graduate School of Chemical Sciences and Engineering, Hokkaido University, Kita 13, Nishi 8, Kita-ku, Sapporo 060-8628, Japan

* Corresponding author E-mail: itoi-hiroyuki@aitech.ac.jp

S1. Electrode preparation and assembly of electrochemical cells

Fig. S1 shows the method to prepare working and counter electrodes and the cell assembly of the three-electrode cell used in this study. An electrode sheet was prepared using carbon black (CB: DENKA BLACK Li, Denka Company Ltd.) and polytetrafluoroethylene (PTFE: PTFE 6-J, Du Pont-Mitsui Fluorochemicals Company, Ltd). In order to prepare working electrodes, CB and PTFE were mixed with AC or the AC/BQD hybrids, and the resulting mixture containing 8.5 mg of AC was weighed. The mixture was formed into a 12×12 mm square sheet and the sheet was sandwiched by SUS304 mesh (100 mesh, Nilaco) at 30 MPa for 150 s. Since BQDs were adsorbed inside the pores of AC without the volume change of AC particles (for details, see section S3), the weight ratio of AC (*i.e.*, excluding the weight of BQD), CB, and PTFE was adjusted to 18:1:1 and the use of the mixture containing 8.5 mg of AC led to the same electrode thickness for the electrodes of AC and the AC/BQD hybrids. The weights of the working electrodes are summarized in Table S1. A counter electrode was prepared using AC (MSC30, Kansai Coke and Chemicals Co., Ltd.), CB, and PTFE, in the same manner as the method to prepare the working electrode, and 20 mg of the mixture was sandwiched by SUS304 mesh. As shown in Fig. S1, a three-electrode beaker cell was prepared using the working and counter electrodes, a Ag/AgCl reference electrode containing saturated aqueous KCl solution, and 15 mL of aqueous 1 M H_2SO_4 electrolyte.

For the two-electrode cell measurements, the same beaker cell was used and the cell voltage (the difference in the potential between the anode and cathode) was controlled in the range from 0 to 0.8 V. To confirm the potentials of the anode and cathode, the Ag/AgCl reference electrode was used during two-electrode cell measurements. An asymmetrical two-electrode cell was prepared using AC/TMBQ as an anode and AC as a cathode. The anode contained 8.5 mg of AC and the weight of the cathode was balanced considering the capacitances of AC/TMBQ and AC measured at 2 A g^{-1} using a three-electrode cell (Fig. 2c). For comparison, a symmetrical two-electrode cell was prepared using AC and both electrodes contained 8.5 mg of AC. The weights of the anodes and cathodes for both asymmetrical and symmetrical cells are summarized in Table S2.

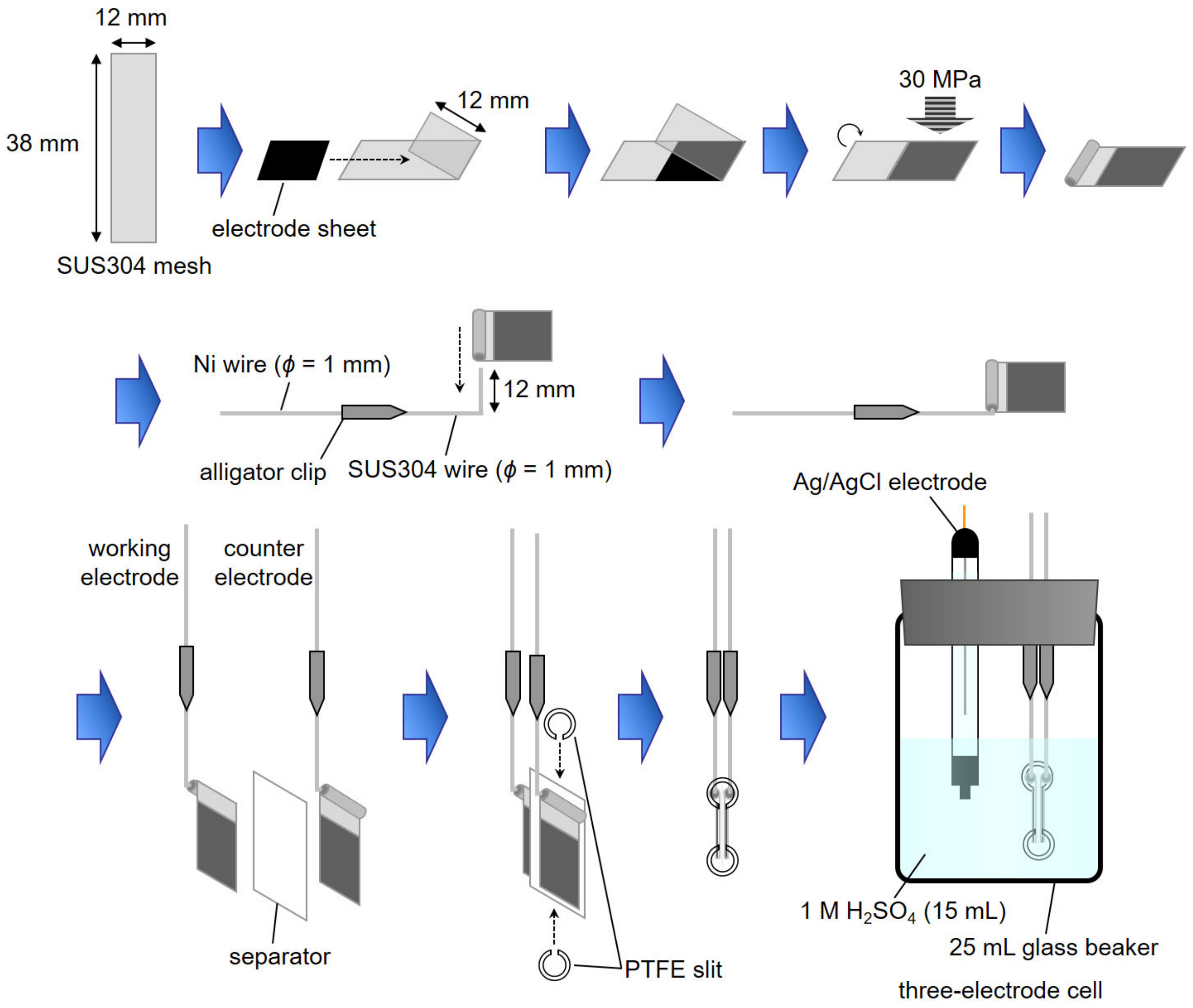

Fig. S1. Schematic illustration for the assembly of the electrochemical cell.

Table S1. Weights of the working electrodes with and without CB and PTFE and the weights of AC in the working electrodes used for the three-electrode cell measurements

Sample	Weights / mg		
	Electrode (with CB and PTFE)	Electrode (without CB and PTFE)	AC
AC	9.306	8.375	8.375
AC/BQ	11.217	10.278	8.452
AC/*m*-DMBQ	11.706	10.772	8.467
AC/TMBQ	12.269	11.324	8.504
AC/^{t}BuBQ	12.291	11.347	8.543
AC/*m*-D^{t}BuBQ	13.118	12.179	8.453
AC/*m*-DCBQ	12.305	11.368	8.400
AC/*p*-DCBQ	12.273	11.344	8.379
AC/TCBQ	13.619	12.676	8.491
AC/*p*-DBrBQ	13.999	13.051	8.507
AC/*p*-DCBQ-L	12.487	11.533	8.500
AC/*p*-DCBQ-L-H	12.224	11.296	8.343
AC/*p*-DCBQ-L#	12.223	11.296	8.343
AC/*p*-DMBQ	11.787	10.844	8.490
AC/*p*-D^{t}BuBQ	13.208	12.264	8.496

Table S2. Gravimetric capacitances measured at 2 A g^{-1} using a three-electrode cell and the weights of the cathodes and anodes used for the two-electrode cell measurements

Cell	Gravimetric capacitance [a]	Weights / mg	
	F g^{-1}	Electrode (with CB and PTFE)	Electrode (without CB and PTFE)
Anode//Cathode	Anode//Cathode	Cathode/Anode	Cathode/Anode
AC//AC	257/257 (1.00)	9.455/9.440	8.509/8.496
AC/TMBQ//AC	440/257 (1.71)	12.265/19.928	11.321/17.935

[a] Gravimetric capacitances measured at 2 A g^{-1} using a three-electrode cell. The values in the parentheses are the ratios in the gravimetric capacitances of the anode and cathode.

S2. Calculations of the volumetric current and the volumetric capacitance in the three-electrode cell measurements

Since 2 mmol of the BQDs were hybridized with 1 g of AC, the weight percentages of the BQDs in the AC/BQD hybrids (X) were calculated using the following equation:

$$\mathrm{X}\,[\%] = \frac{M_{\mathrm{BQD}}[\mathrm{g}]}{M_{\mathrm{BQD}}[\mathrm{g}] + 1[\mathrm{g}]} \times 100 = \frac{2 \times 10^{-3}[\mathrm{mol}] \times MW_{\mathrm{BQD}}[\mathrm{g\,mol^{-1}}]}{2 \times 10^{-3}[\mathrm{mol}] \times MW_{\mathrm{BQD}}[\mathrm{g\,mol^{-1}}] + 1[\mathrm{g}]} \times 100 \quad (1)$$

where $M_{\mathrm{BQD}}[\mathrm{g}]$ and $MW_{\mathrm{BQD}}[\mathrm{g\,mol^{-1}}]$ are the weight of 2 mmol of BQD and the molecular weight of BQD, respectively. Therefore, the weight ratio of AC and BQDs in the AC/BQD hybrids is $(100 - \mathrm{X})\!:\mathrm{X}$. As shown in Fig. S2a, the electrode of AC consists of AC, binder (*i.e.*, PTFE), and CB, and the weights [g] of AC, PTFE, and CB per 1 cm^3 of the electrode are defined as W_{AC}, W_{PTFE}, and W_{CB}, respectively. The experimental electrode density of AC is defined as ρ_{AC}^{ex} $[\mathrm{g\,cm^{-3}}]$ and equal to the sum of W_{AC}, W_{PTFE}, and W_{CB}. Meanwhile, assuming that the adsorption of BQD is not accompanied by the volume change of the AC particles (for details, see Section S3), the weights of 1 cm^3 of the electrodes for the AC/BQD hybrids are equal to the sum of W_{AC}, W_{PTFE}, W_{CB}, and W_{BQD} (Fig. S2b), where W_{BQD} is the weight of BQD per 1 cm^3 of the electrode. For the electrodes of both AC and the AC/BQD hybrids, the weight ratio of W_{AC}, W_{PTFE}, and W_{CB} is 18:1:1; therefore, W_{AC} is equal to $0.9\rho_{\mathrm{AC}}^{ex}$, and W_{PTFE} and W_{CB} are equal to $0.05\rho_{\mathrm{AC}}^{ex}$. Meanwhile, the weight ratios of W_{AC}, W_{BQD}, W_{PTFE}, and W_{CB} in the electrodes of the AC/BQD hybrids are 18:18X/$(100 - \mathrm{X})$:1:1. The current per 1 g of AC in the AC/BQD hybrids ($I_{\mathrm{g-AC}}$ $[\mathrm{A\,g_{AC}^{-1}}]$) was calculated using the current per 1 g of the AC/BQD hybrids excluding the weights of CB and PTFE (I_{g} $[\mathrm{A\,g^{-1}}]$), based on the following equation:

$$I_{\mathrm{g-AC}}\,[\mathrm{A\,g_{AC}^{-1}}] = I_{\mathrm{g}}\,[\mathrm{A\,g^{-1}}] \times \frac{100}{100 - \mathrm{X}} \quad (1)$$

The volumetric current of AC and the AC/BQD hybrids (I_{v} $[\mathrm{A\,cm^{-3}}]$: the current per 1 cm^3 of the electrode) was calculated according the following equation:

$$I_{\mathrm{v}}\,[\mathrm{A\,cm^{-3}}] = I_{\mathrm{g-AC}}\,[\mathrm{A\,g_{AC}^{-1}}] \times W_{\mathrm{AC}}\,[\mathrm{g_{AC}\,cm^{-3}}] = I_{\mathrm{g}}\,[\mathrm{A\,g^{-1}}] \times \frac{100}{100 - \mathrm{X}} \times 0.9\rho_{\mathrm{AC}}^{ex}\,[\mathrm{g\,cm^{-3}}]$$

$$= \frac{90 I_{\mathrm{g}} \rho_{\mathrm{AC}}^{ex}}{100 - \mathrm{X}} \quad (2)$$

As well as the volumetric current, the volumetric capacitance can be similarly calculated according to the following equations:

$$C_{\mathrm{g-AC}}\ [\mathrm{F\ g_{AC}^{-1}}] = C_{\mathrm{g}}\ [\mathrm{F\ g^{-1}}] \times \frac{100}{100 - \mathrm{X}} \qquad (3)$$

$$C_{\mathrm{v}}\ [\mathrm{F\ cm^{-3}}] = C_{\mathrm{g-AC}}\ [\mathrm{F\ g_{AC}^{-1}}] \times W_{\mathrm{AC}}\ [\mathrm{g_{AC}\ cm^{-3}}] = C_{\mathrm{g}}\ [\mathrm{F\ g^{-1}}] \times \frac{100}{100 - \mathrm{X}} \times 0.9\rho_{\mathrm{AC}}^{ex}\ [\mathrm{g\ cm^{-3}}]$$

$$= \frac{90 C_{\mathrm{g}} \rho_{\mathrm{AC}}^{ex}}{100 - \mathrm{X}} \qquad (4)$$

The areal capacitance (C_{A} [F cm^{-2}]) was calculated using the area of the working electrode (1.44 cm^2, Fig. S1), according to the following equation:

$$C_{\mathrm{A}}\ [\mathrm{F\ cm^{-2}}] = C_{\mathrm{g}}\ [\mathrm{F\ g^{-1}}] \times \frac{(w_{\mathrm{AC}} + w_{\mathrm{PTFE}} + w_{\mathrm{CB}} + w_{\mathrm{BQD}})[\mathrm{g}]}{1.44\ [\mathrm{cm^2}]} \qquad (5)$$

where w_{AC}, w_{PTFE}, w_{CB}, and w_{BQD} are the weights of AC, PTFE, CB, and BQD in the electrode, respectively. The gravimetric capacity (C'_{g} [mA h g^{-1}]) was calculated using the following equation:

$$C'_{\mathrm{g}}\ [\mathrm{mA\ h\ g^{-1}}] = \frac{1000 \times I\ [\mathrm{mA}] \times \Delta t'\ [\mathrm{h}]}{(w_{\mathrm{AC}} + w_{\mathrm{PTFE}} + w_{\mathrm{CB}} + w_{\mathrm{BQD}})\ [\mathrm{g}]} = C_{\mathrm{g}}\ [\mathrm{F\ g^{-1}}] \times \frac{1000\ [\mathrm{mA\ A^{-1}}]}{3600\ [\mathrm{s\ h^{-1}}]} \times 0.9[\mathrm{V}]$$

$$= 0.25 C_{\mathrm{g}} \qquad (6)$$

where Δ*t*’ is the time [h] from −0.1 to 0.8 V, and 0.9 V corresponds to the potential window. Similarly, the volumetric capacity (C'_{v} [mA h cm^{-3}]) and the areal capacity (C'_{A} [mA h cm^{-2}]) were calculated using the following equations:

$$C'_{\mathrm{v}}\ [\mathrm{mA\ h\ cm^{-3}}] = 0.25 C_{\mathrm{v}} \qquad (7)$$

$$C'_{\mathrm{A}}\ [\mathrm{mA\ h\ cm^{-2}}] = 0.25 C_{\mathrm{A}} \qquad (8)$$

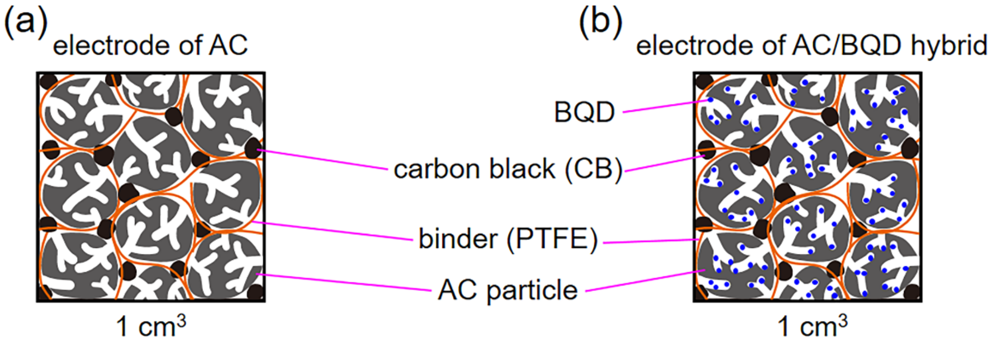

(a)

$$W_{\mathrm{AC}}:W_{\mathrm{CB}}:W_{\mathrm{PTFE}} = 18:1:1$$

$$W_{\mathrm{AC}} = 0.9\rho_{\mathrm{AC}}^{ex}$$

$$W_{\mathrm{CB}} = W_{\mathrm{PTFE}} = 0.05\rho_{\mathrm{AC}}^{ex}$$

$$\rho_{\mathrm{AC}}^{ex} = W_{\mathrm{AC}} + W_{\mathrm{CB}} + W_{\mathrm{PTFE}}$$

(b)

$$W_{\mathrm{AC}}:W_{\mathrm{CB}}:W_{\mathrm{PTFE}} = 18:1:1$$

$$W_{\mathrm{AC}}:W_{\mathrm{BQD}} = (100 - \mathrm{X}):\mathrm{X}$$

$$W_{\mathrm{AC}}:W_{\mathrm{BQD}}:W_{\mathrm{CB}}:W_{\mathrm{PTFE}} = 18:\frac{18\mathrm{X}}{100 - \mathrm{X}}:1:1$$

$$W_{\mathrm{AC}} = 0.9\rho_{\mathrm{AC}}^{ex}$$

$$W_{\mathrm{CB}} = W_{\mathrm{PTFE}} = 0.05\rho_{\mathrm{AC}}^{ex}$$

$$W_{\mathrm{BQD}} = 0.9\rho_{\mathrm{AC}}^{ex}\frac{\mathrm{X}}{(100 - \mathrm{X})}$$

Fig. S2. Schematic of the electrodes of (a) AC and (b) AC/BQD hybrids.

S3. Calculation of the electrode densities of the AC/BQD hybrids

In our previous studies, we have reported that the adsorption of organic and organometallic compounds was not accompanied by the volume change of porous carbon particles because the experimental electrode densities are in good agreement with the theoretical ones.[1-6] Specifically, in the case of using p-DCBQ (2,5-dichloro-1,4-benzoquinone) and the same AC used in this study, the experimental electrode densities were the same as those of the theoretical ones despite the amount of p-DCBQ, where the amount of p-DCBQ in the AC/p-DCBQ hybrids was examined up to the saturation amount: *i.e.*, 14.0 mmol per 1 g of AC (71.2 wt%).[4] Therefore, we used the theoretical electrode densities in this study. As shown in Fig. S2, the theoretical electrode densities (ρ^{theo} [g cm^{-3}]) can be calculated as the sum of W_{AC}, W_{BQD}, W_{PTFE}, and W_{CB}, according to the following equation:[3, 7]

$$\rho^{theo}\ [\mathrm{g\ cm^{-3}}] = W_{\mathrm{AC}} + W_{\mathrm{PTFE}} + W_{\mathrm{CB}} + W_{\mathrm{BQD}} = \rho_{\mathrm{AC}}^{ex} + 0.9\rho_{\mathrm{AC}}^{ex}\frac{\mathrm{X}}{100-\mathrm{X}} = \rho_{\mathrm{AC}}^{ex}\left(\frac{\mathrm{X}}{100-\mathrm{X}} + 0.1\right) \quad (9)$$

The experimental electrode density of AC is 0.33 g cm^{-3}.[4] The electrode densities of AC/p-DCBQ-L, AC/p-DCBQ-L-H, and AC/p-DCBQ-L# are regarded as the same values as that of AC/p-DCBQ.

Table S3. Weight percentages of BQDs (X) and theoretical electrode densities of the AC/BQD hybrids (ρ^{theo}).

samples	X / wt%	ρ^{theo} / g cm^{-3}
AC/BQ	17.8	0.39
AC/*m*-DMBQ	21.4	0.41
AC/TMBQ	24.7	0.42
AC/^{t}BuBQ	24.7	0.42
AC/*m*-D^{t}BuBQ	30.6	0.46
AC/*m*-DCBQ	26.1	0.43
AC/*p*-DCBQ	26.1	0.43
AC/TCBQ	33.0	0.47
AC/*p*-DBrBQ	34.7	0.48
AC/*p*-DMBQ	21.4	0.41
AC/*p*-D^{t}BuBQ	30.6	0.46

S4. TG-DTA analyses of BQDs

To determine the adsorption temperatures of BQDs, thermogravimetry-differential thermal analysis (TG-DTA) was performed on Simultaneous Thermogravimetry/Differential Thermal Analyzer (DTG-60, Shimadzu). BQDs were measured at a ramp rate of 10 ºC min^{-1} up to 500 ºC under nitrogen gas (100 mL min^{-1}). The results are shown in Fig. S3. The melting point of *m*-DCBQ is 122 ºC and *m*-DCBQ shows a distinct peak at *ca*. 120 ºC (Fig. S3a), corresponding to fusion. However, the weight of *m*-DCBQ decreases below its melting temperature due to sublimation. Therefore, in order to perform gas-phase adsorption, the adsorption temperature of *m*-DCBQ was set to 105 ºC. Similarly, the adsorption temperatures of the other BQDs were determined, as summarized in Table S4.

Table S4. Melting points and adsorption temperatures of BQDs

BQDs	Melting point / ºC	Adsorption temperature / ºC
BQ	113	25
m-DMBQ	73	65
TMBQ	112	100
^{t}BuBQ	57	55
m-D^{t}BuBQ	67	60
m-DCBQ	122	105
p-DCBQ	161	100
TCBQ	290	200
p-DBrBQ	193	160
p-DMBQ	125	115
p-D^{t}BuBQ	153	140

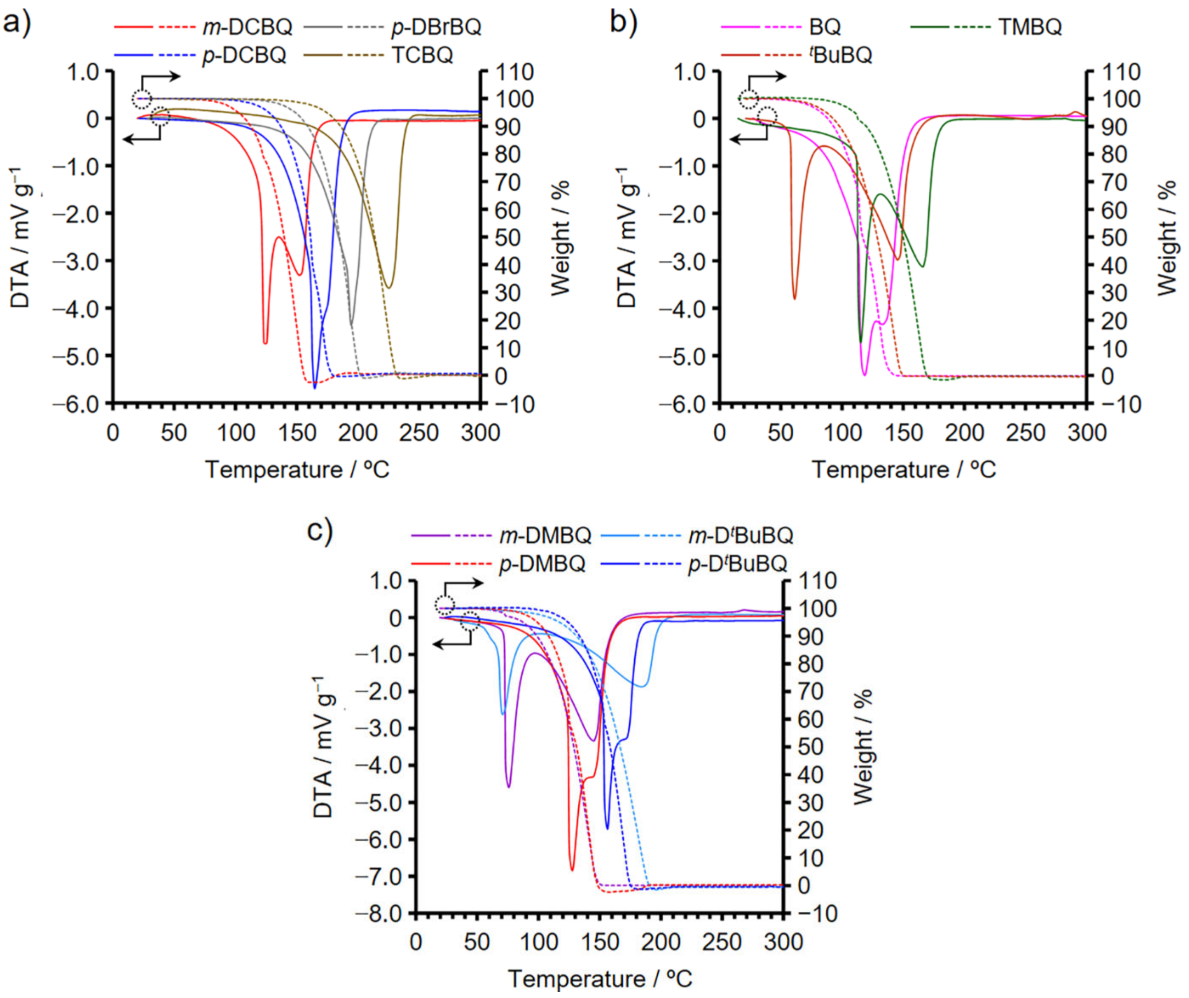

Fig. S3. TG-DTA patterns of BQDs: (a) *m*-DCBQ, *p*-DCBQ, *p*-DBrBQ, and TCBQ; (b) BQ, TMBQ, and ^{t}BuBQ; (c) *m*-DMBQ, *p*-DMBQ, *m*-D^{t}BuBQ, and *p*-D^{t}BuBQ.

S5. XRD patterns of AC, the AC/BQD hybrids, and BQDs

Fig. S4a shows the XRD patterns of the AC/ABQ hybrids, AC/BQ, ABQs, and BQ. Meanwhile, Fig. S4b shows the XRD patterns of AC, the AC/HBQ hybrids, and HBQs. The distinct peaks in bulk BQDs are based on their crystalline structures. When BQDs are adsorbed inside the pores of AC, they cannot retain the same crystalline structures as those in the bulk because the pore size of AC is ~4 nm and the size and structure of adsorbed BQDs are restricted inside such small pores. The peaks derived from the crystalline structures in the XRD patterns become broad as their particle sizes become small, as shown in well-known Scherrer equation:[8]

$$L = K\lambda/\beta\cos\theta$$

where L is the mean crystal size (nm), K is the Scherrer constant depending on the crystallite shape normally taken as 0.9, λ is the X-ray wavelength (0.154 nm for Cu Kα), β is the full width of the diffracted peak profile at half-maximum height (in radians 2θ), and θ is the Bragg reflection angle in radians. Therefore, very small particles do not show distinct peaks in the XRD pattern. All hybrids do not show any distinct peaks derived from the crystalline BQDs but show a broad peak in their XRD patterns; *i.e.*, the BQD molecules exist inside the pores of AC and there are few BQD molecules on the particle surface of AC.

Since the positions of the broad peaks in the XRD patterns of the AC/BQD hybrids have nothing to do with those of the bulk BQDs, the positions of the broad peaks would be related to the distance between the BQD molecules and the carbon pore surfaces, which are interacted through π-π stacking. If this is the case, the distance must have positive correlation with the size of functional groups on the BQD molecules; large functional groups distance BQD molecules from the carbon surface. The distance between the carbon surface and benzene interacted through π-π stacking is reported to be 0.324 nm.[9] Meanwhile, tetrafluoro-1.4-benzoquinone interacts with phenanthrene through π-π stacking and the distance is *ca*. 0.33 nm.[10] The interaction between phenanthrene and tetrafluoro-1.4-benzoquinone is similar to that of the BQDs and carbon surfaces for the AC/BQD hybrids and the distance of 0.33 nm corresponds to 27° (2θ). The bulky functional groups in BQD molecules further distance the molecules from the carbon surface, resulting in the shift of the broad peak to the low angle in the XRD pattern, which is observed for the hybrids of bulky ABQs: *e.g.*, AC/^{t}BuBQ and AC/p-D^{t}BuBQ.

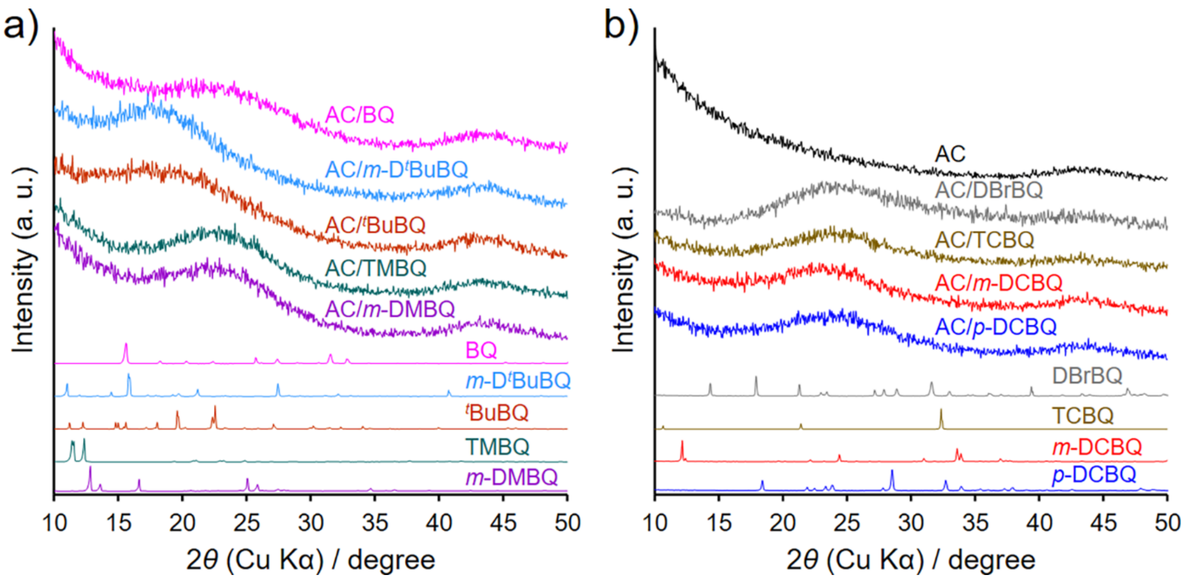

Fig. S4. (a and b) XRD patterns of (a) ABQs, BQ, AC/BQ, and AC/ABQ hybrids and (b) HBQs, AC/HBQ hybrids, and AC.

S6. Results of nitrogen adsorption/desorption measurements for AC and the AC/BQD hybrids

The nitrogen adsorption/desorption isotherms and pore size distributions of AC, AC/BQ, and the AC/ABQ hybrids are shown in Fig. S5a and b, respectively. Fig. S5c and d show the nitrogen adsorption/desorption isotherms and pore size distributions, respectively, for AC and the AC/HBQ hybrids. The amount of adsorbed nitrogen is normalized per 1 g of AC in Fig. S5. The nitrogen adsorption/desorption isotherms of AC and all the hybrids show the steep uptake below P/P_0 of *ca*. 0.05 due to the micropore filling. In addition, a gradual increase of adsorbed nitrogen is observed up to P/P_0 of *ca*. 0.4 without hysteresis. The steep uptake is based on the micropores while the gradual increase without hysteresis is derived from small mesopores. Therefore, the type of the isotherms is the combination of types I and IV. Table S5 summarizes the BET surface areas and pore volumes of AC and the AC/BQD hybrids, shown alongside the normalized values. The normalized values of the BET surface areas and pore volumes decrease with increasing the molecular sizes of the BQDs. In addition, both micropore and mesopore volumes are decreased by the hybridization of BQDs (Table S5), indicating that BQD molecules do not localize in the micropores or mesopores.

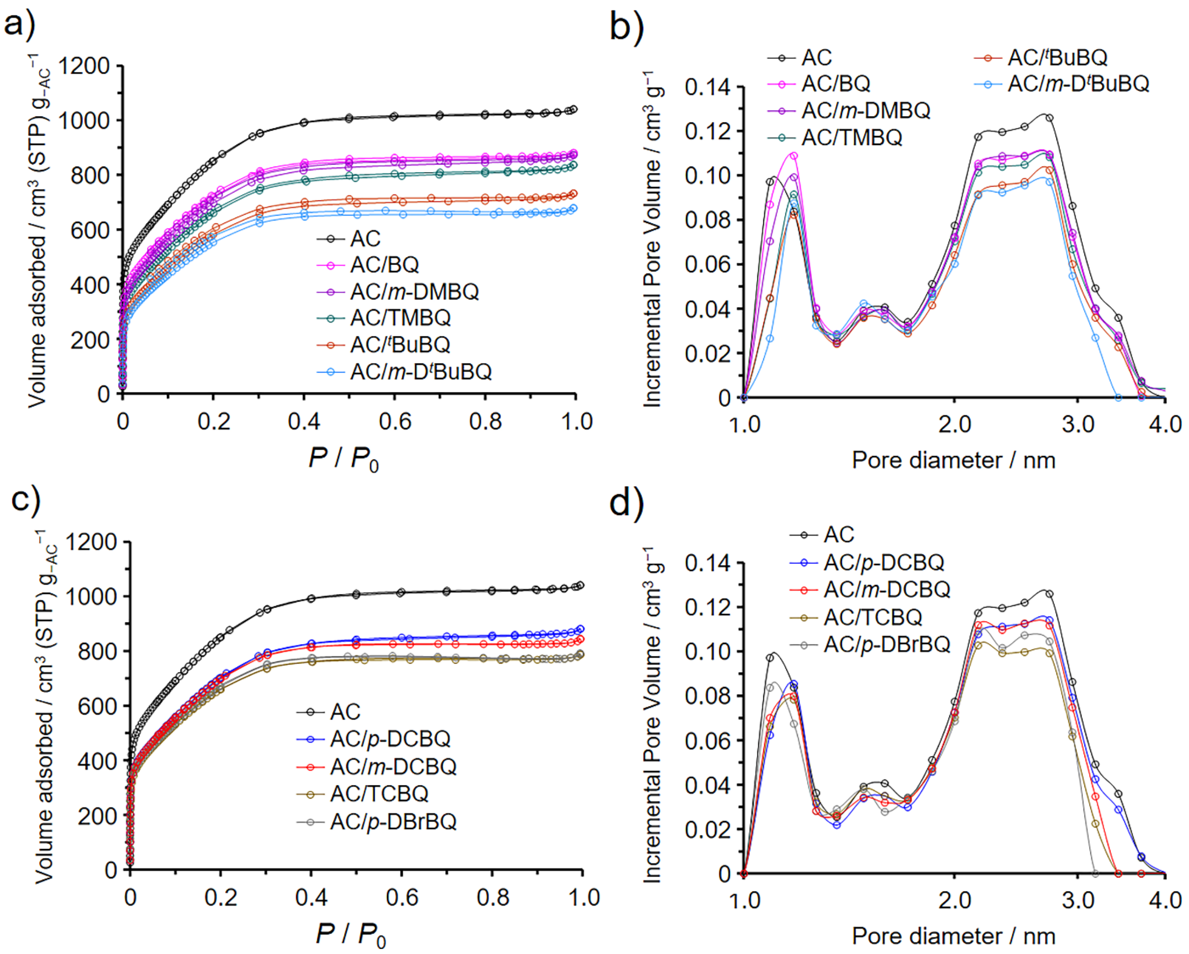

Fig. S5. Nitrogen adsorption/desorption isotherms (a, c) and pore size distributions (b, d): (a, b) AC, AC/BQ, and the AC/ABQ hybrids; (c, d) AC and the AC/HBQ hybrids. The pore size distributions are calculated by the density functional theory (DFT) method based on the carbon slit pores (77 K, N_2).

Table S5. BET surface areas and pore volumes of AC and AC/BQD hybrids.

Samples	wt%	S_{BET} [a)] [m^2 g^{-1}]	V_{total} [cm^3 g^{-1}]	V_{micro} [cm^3 g^{-1}]	V_{meso} [cm^3 g^{-1}]	S_{BET} [b)] [m^2 g^{-1}]	V_{total} [b)] [cm^3 g^{-1}]	V_{micro} [b)] [cm^3 g^{-1}]	V_{meso} [b)] [cm^3 g^{-1}]
AC	-	3160	1.59	0.99	0.60				
AC/*m*-DMBQ	21.4	2060	1.04	0.63	0.42	2620	1.33	0.80	0.53
AC/TMBQ	24.7	1880	0.95	0.56	0.39	2500	1.27	0.74	0.53
AC/^{t}BuBQ	24.7	1660	0.83	0.49	0.34	2210	1.11	0.66	0.45
AC/*m*-D^{t}BuBQ	30.6	1460	0.71	0.43	0.28	2110	1.02	0.63	0.40
AC/BQ	17.8	2220	1.11	0.69	0.41	2700	1.34	0.84	0.50
AC/*p*-DCBQ	26.1	1960	0.99	0.58	0.40	2650	1.34	0.79	0.54
AC/*m*-DCBQ	26.1	1940	0.95	0.58	0.36	2630	1.28	0.79	0.49
AC/*p*-DBrBQ	34.7	1640	0.78	0.50	0.28	2520	1.19	0.77	0.43
AC/TCBQ	33.0	1660	0.80	0.51	0.29	2470	1.19	0.76	0.44

[a)] BET surface area; [b)] Normalized values per 1 g of AC.

S7. SEM observation of AC and the AC/BQD hybrids

The scanning electron microscopy (SEM) images were collected on JCM-7000 NeoScope (JEOL) at an accelerating voltage of 15 kV. The SEM images of AC and the AC/BQD hybrids are shown in Fig. S6−S8. By comparing with the SEM images of AC (Fig. S6a−c), a difference in morphology between AC and the AC/BQD hybrids was not observed in the SEM images. Considering the results of the XRD and nitrogen adsorption/desorption analyses, there is few BQD molecules on the particle surface of AC for the AC/BQD hybrids.

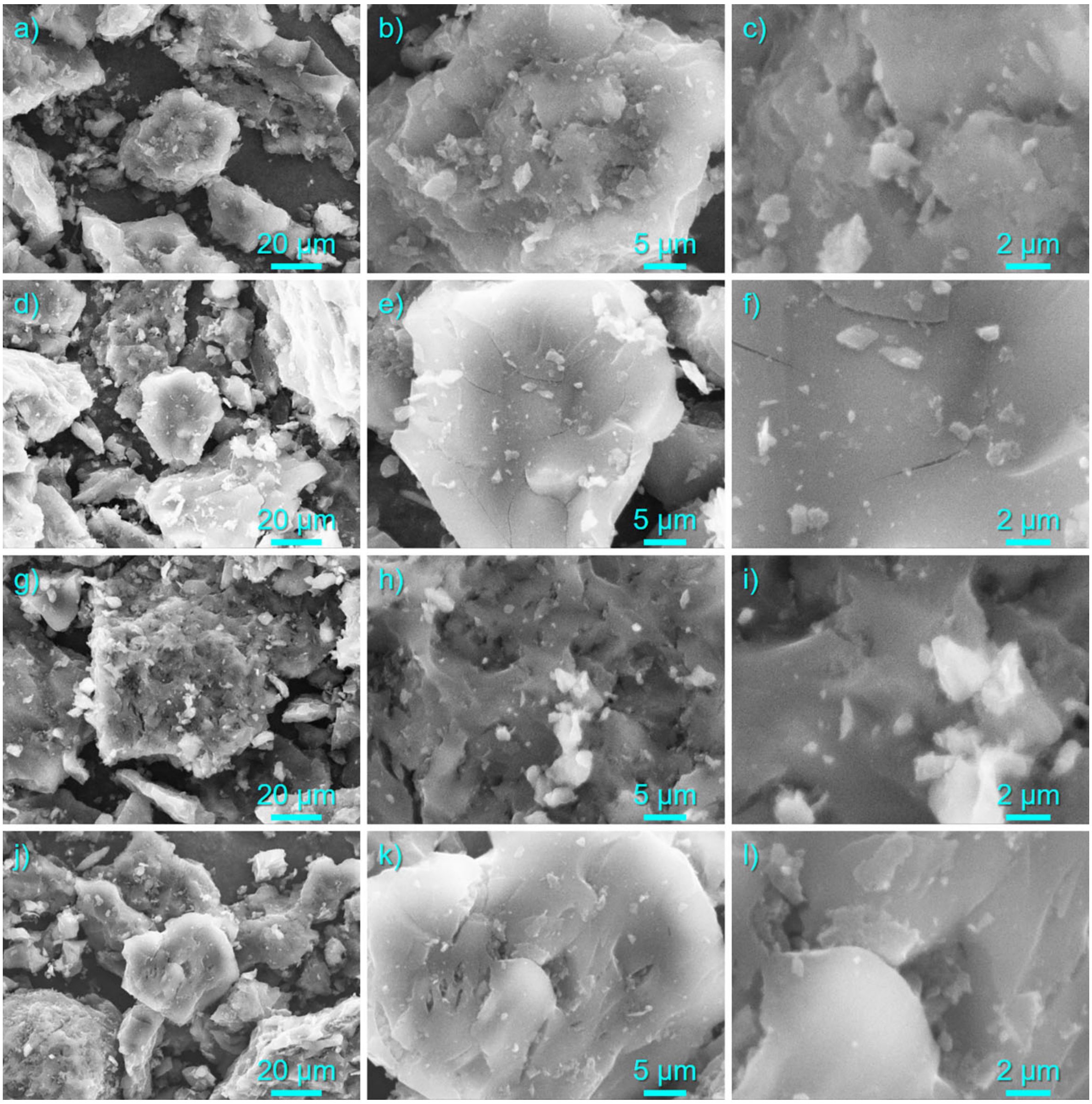

Fig. S6. SEM images of (a−c) AC, (d−f) AC/BQ, (g−i) AC/*m*-DMBQ, and (j−l) AC/TMBQ.

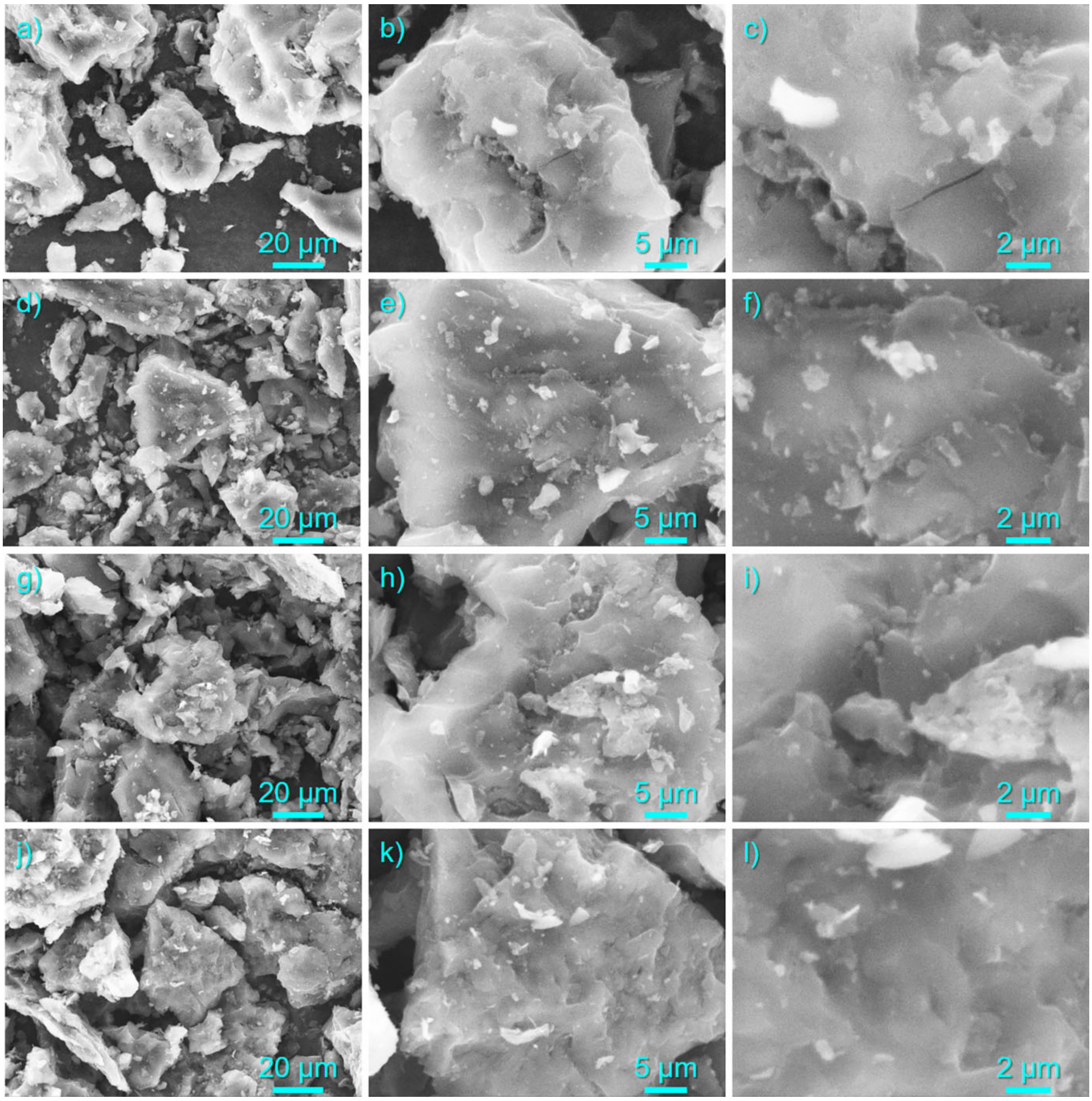

Fig. S7. SEM images of (a–c) AC/^{t}BuBQ, (d–f) AC/*m*-D^{t}BuBQ, (g–i) AC/*m*-DCBQ, and (j–l) AC/*p*-DCBQ.

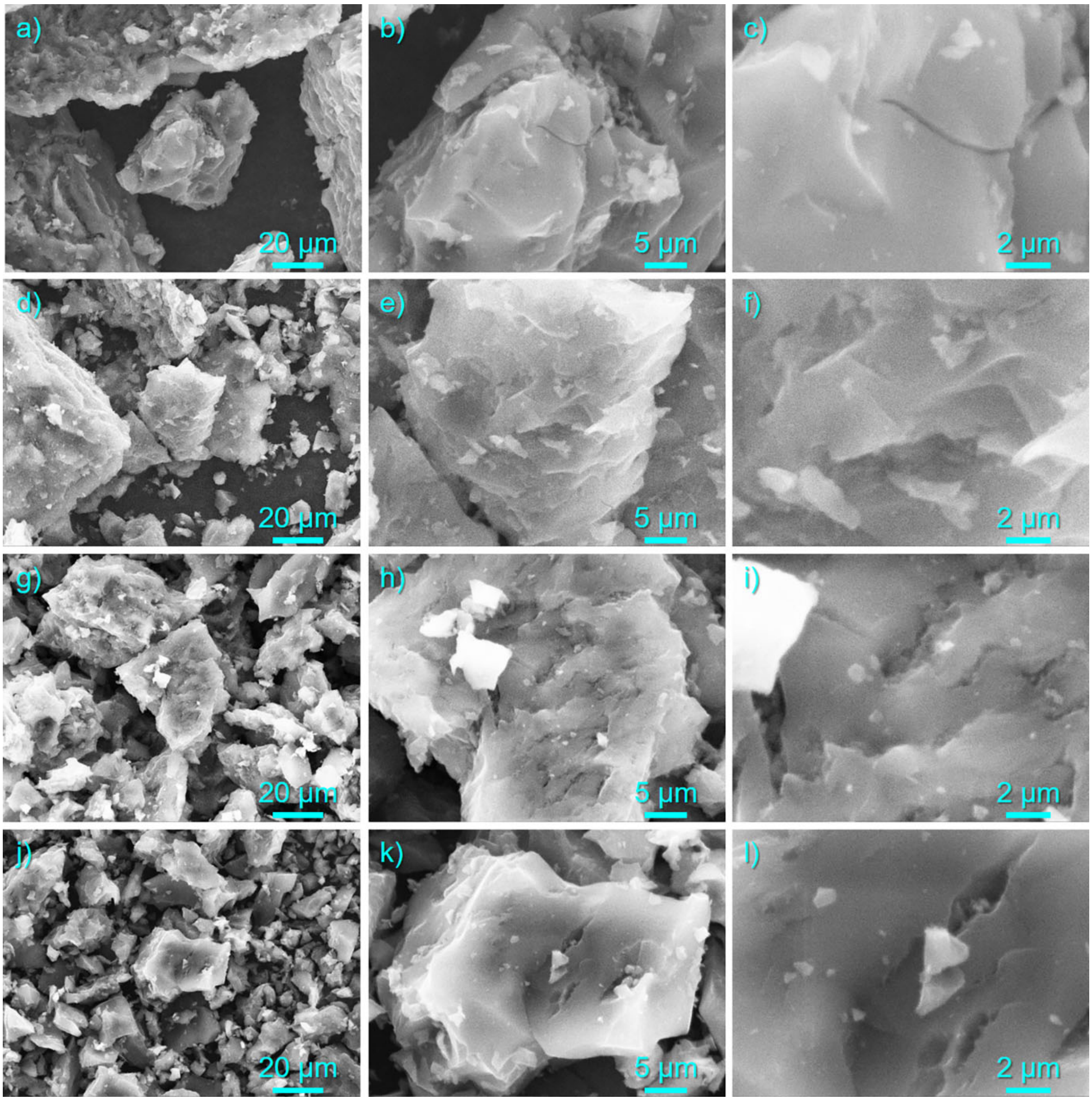

Fig. S8. SEM images of (a–c) AC/TCBQ, (d–f) AC/*p*-DBrBQ, (g–i) AC/*p*-DCBQ-L-H, and (j–l) AC/*p*-DCBQ-L#.

S8. Raman spectra of AC and the AC/BQD hybrids.

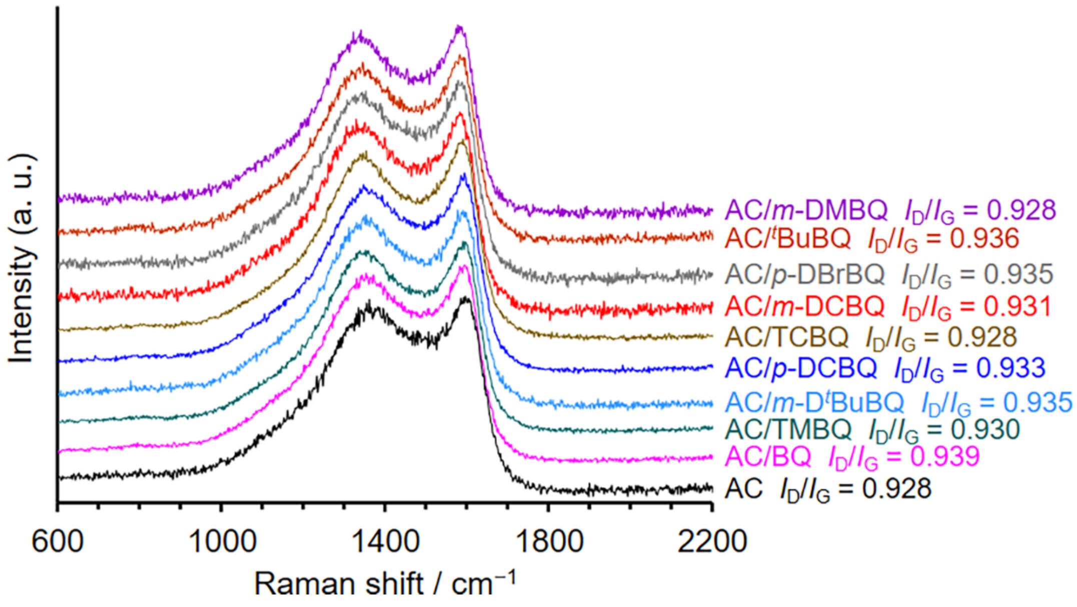

Fig. S9. Raman spectra of AC and AC/BQD hybrids (Laser line: 532 nm).

S9. A method to calculate the utilization efficiencies of the BQDs

The utilization efficiencies of the BQDs were calculated by integrating the quantity of electricity in the anodic peak area, as shown in Fig. S10. The voltammograms collected at 1 mV s^{-1} were used for the calculation. First, a baseline was drawn by extrapolating the constant current, which is attributed to the formation of the electric double-layer. Then, the peak current was integrated, as indicated in the figure. The calculated quantity of current was then compared with the theoretical value, which was calculated considering that all the hybridized BQD molecules underwent reversible redox reactions based on the two-electron redox reaction.

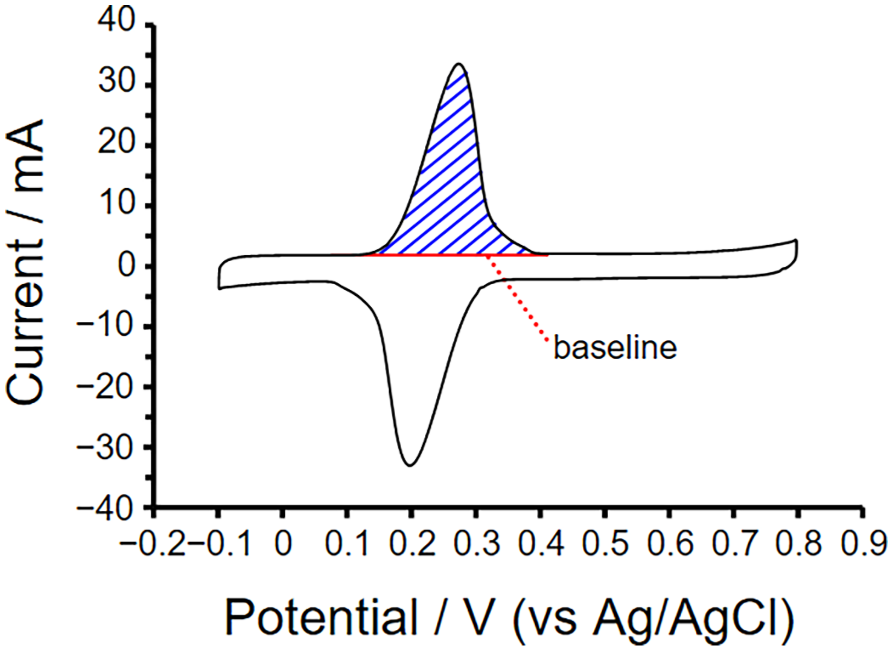

Fig. S10. Calculation of the utilization efficiencies of the BQDs in the AC/BQD hybrids using a cyclic voltammogram collected at 1 mV s^{-1}.

S10. GC curves of AC and the AC/BQD hybrids

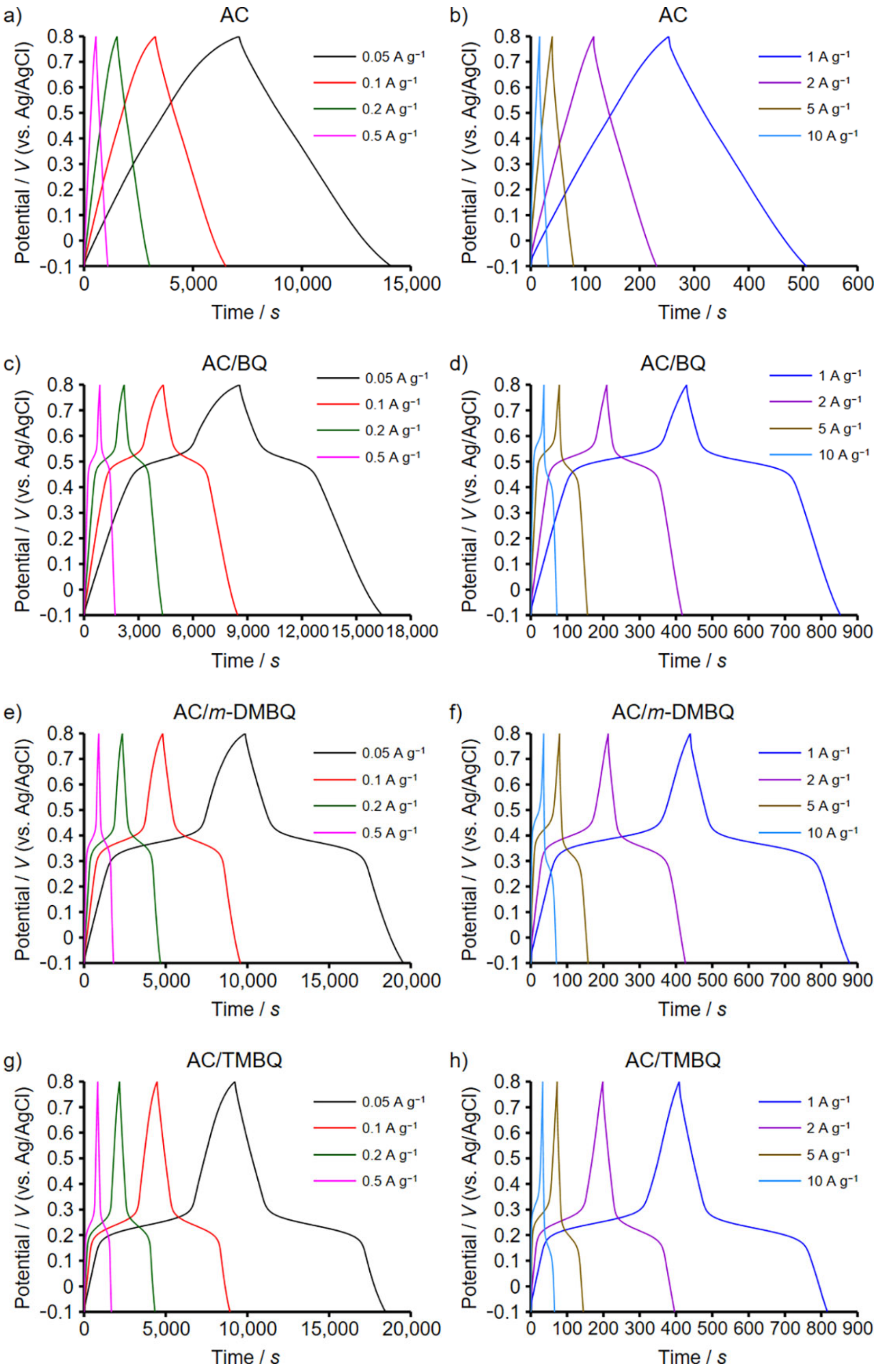

Fig. S11. GC curves for (a, b) AC, (c, d) AC/BQ, (e, f) AC/*m*-DMBQ, and (g, h) AC/TMBQ.

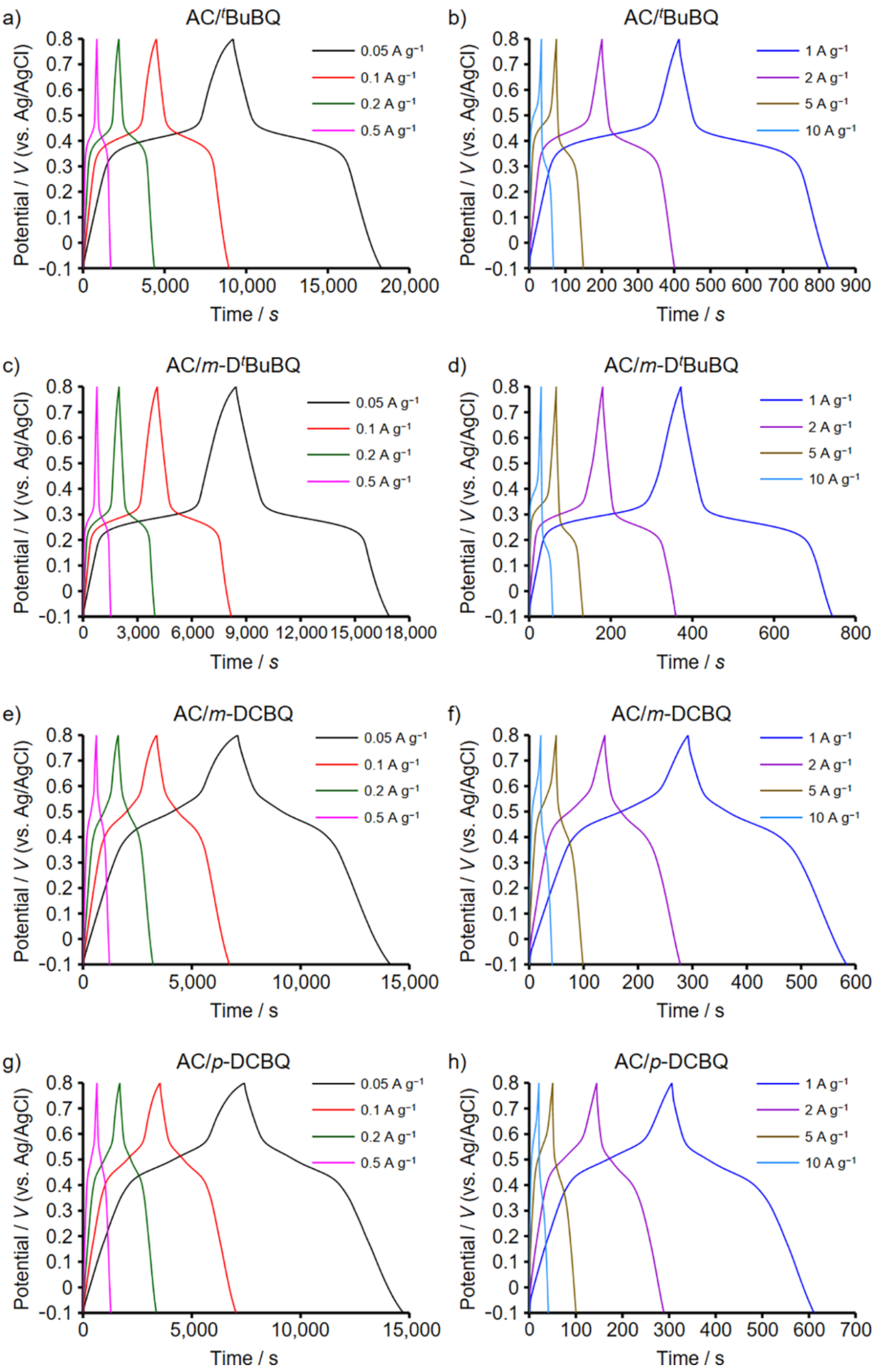

Fig. S12. GC curves for (a, b) AC/^{t}BuBQ, (c, d) AC/*m*-D^{t}BuBQ, (e, f) AC/*m*-DCBQ, and (g, h) AC/*p*-DCBQ.

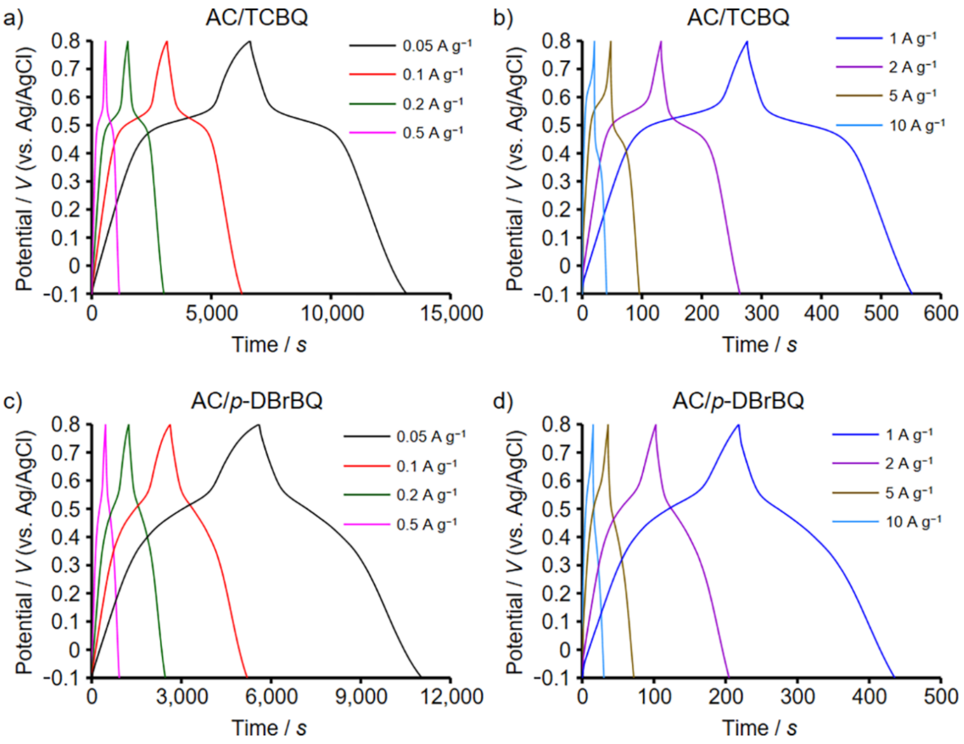

Fig. S13. GC curves for (a, b) AC/TCBQ and (c, d) AC/*p*-DBrBQ.

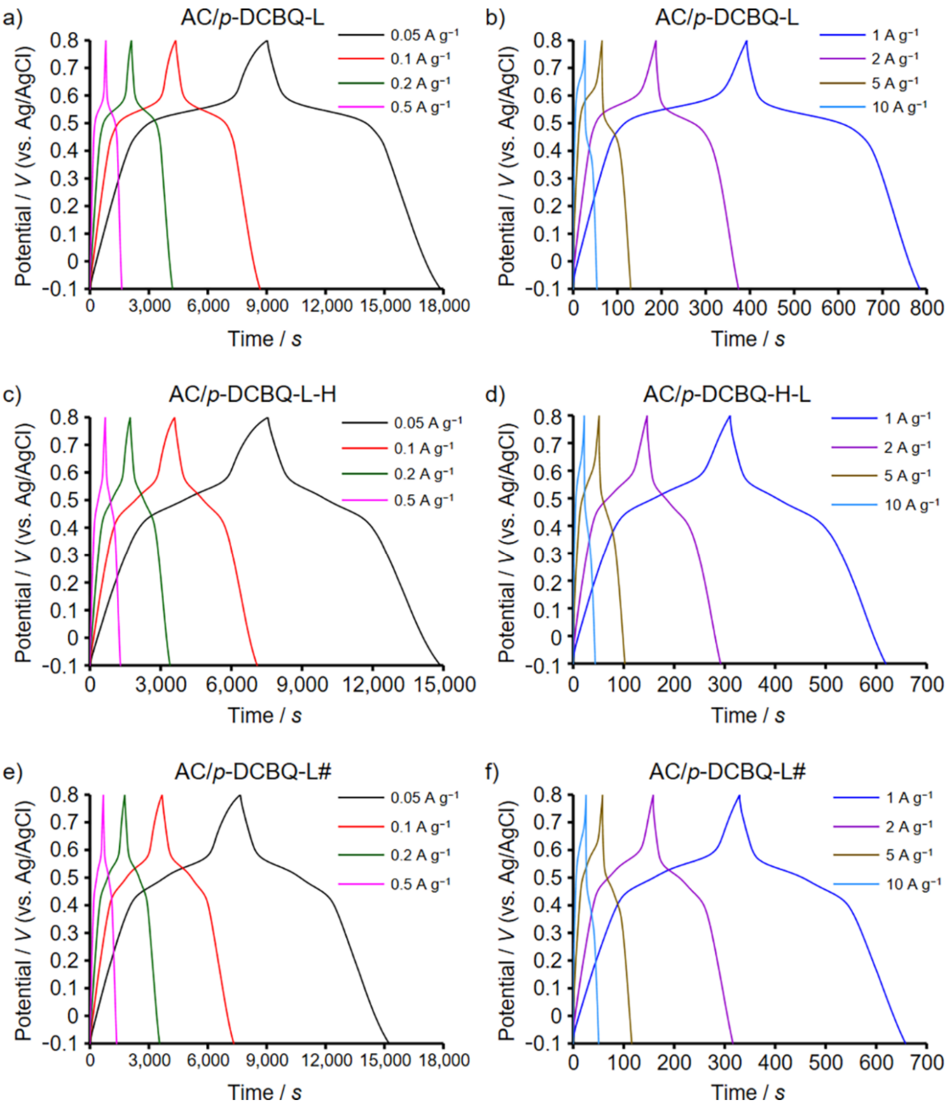

Fig. S14. GC curves for (a, b) AC/*p*-DCBQ-L, (c, d) AC/*p*-DCBQ-L-H, and (e, f) AC/*p*-DCBQ-L#.

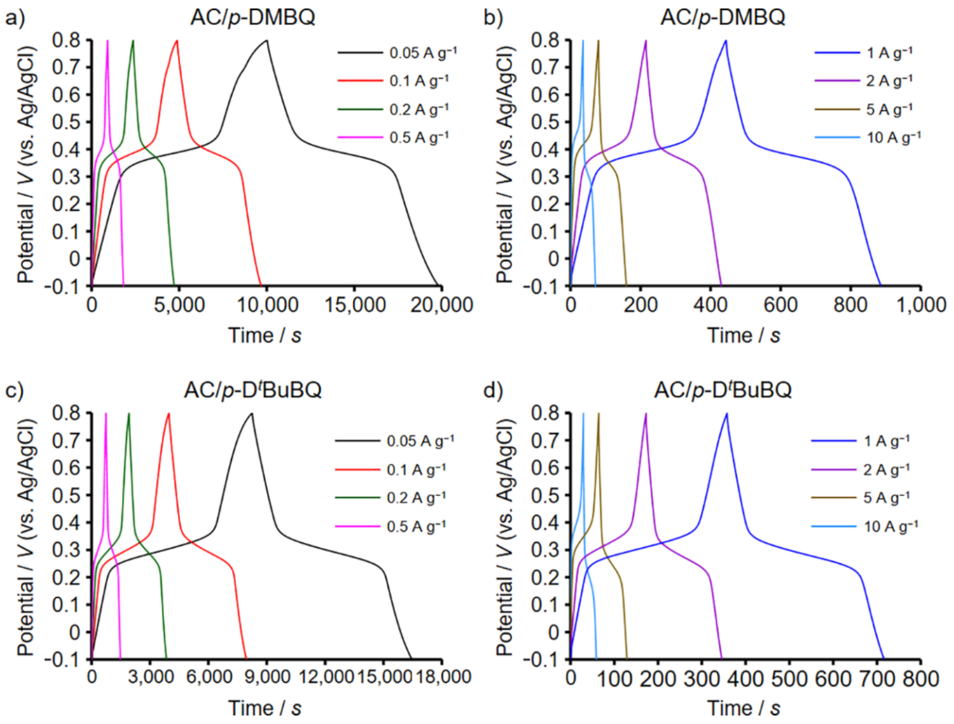

Fig. S15. GC curves for (a, b) AC/*p*-DMBQ and (c, d) AC/*p*-D^{t}BuBQ.

S11. Areal capacitances and gravimetric, volumetric, and areal capacities of AC and the AC/BQD hybrids

The areal capacitances of AC and the AC/BQD hybrids are shown in Fig. S16a. Their gravimetric, volumetric, and areal capacities are shown in Fig. S16b, c, and d, respectively.

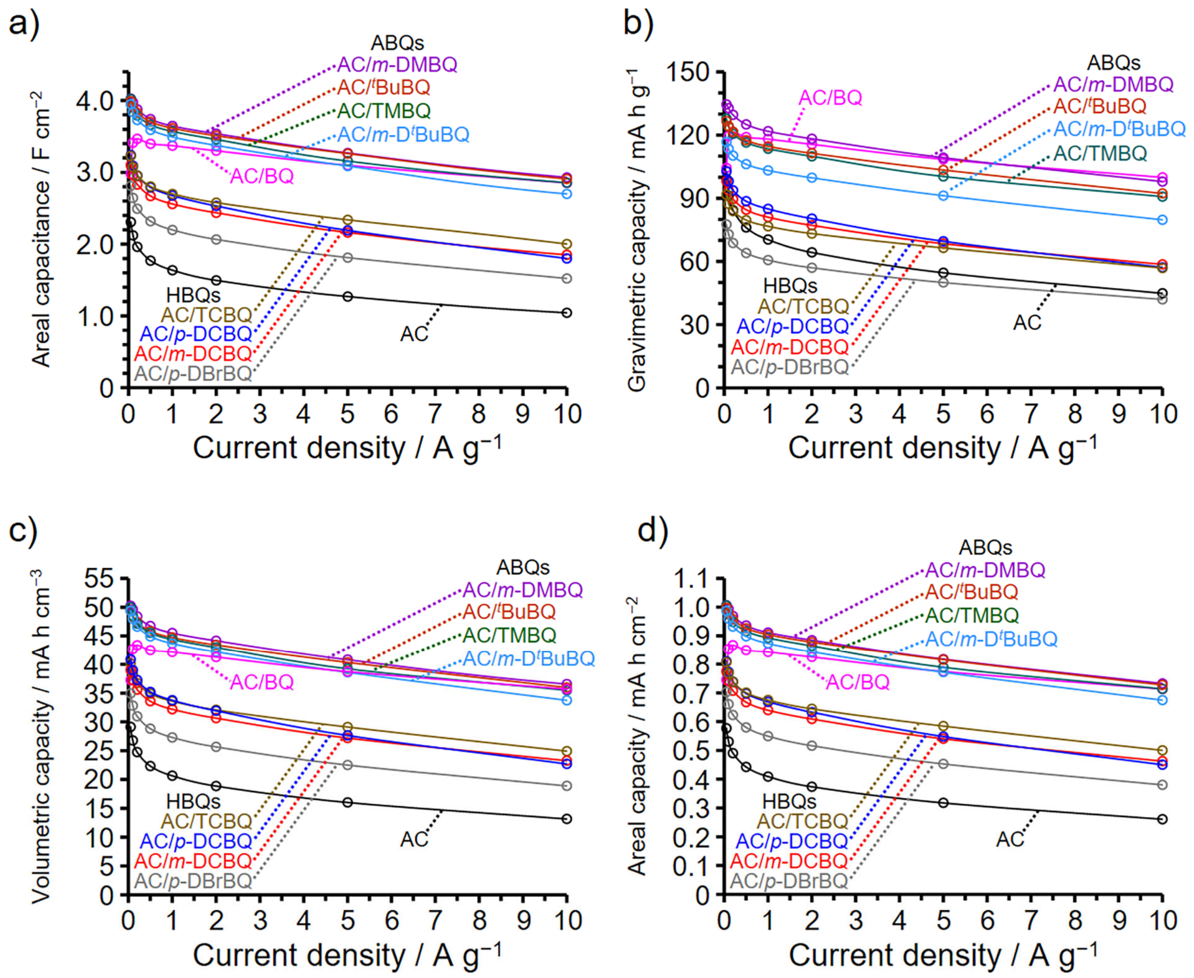

Fig. S16. Results of electrochemical measurements performed at 25 ºC using aqueous 1 M H_2SO_4 electrolyte and a three-electrode cell for AC and the AC/BQD hybrids. (a) Areal capacitances. (b−d) (b) Gravimetric, (c) volumetric, and (d) areal capacities.

S12. Gravimetric and areal capacitances and gravimetric, volumetric, and areal capacities of AC/*p*-DCBQ-L, AC/*p*-DCBQ-L-H, and AC/*p*-DCBQ-L#

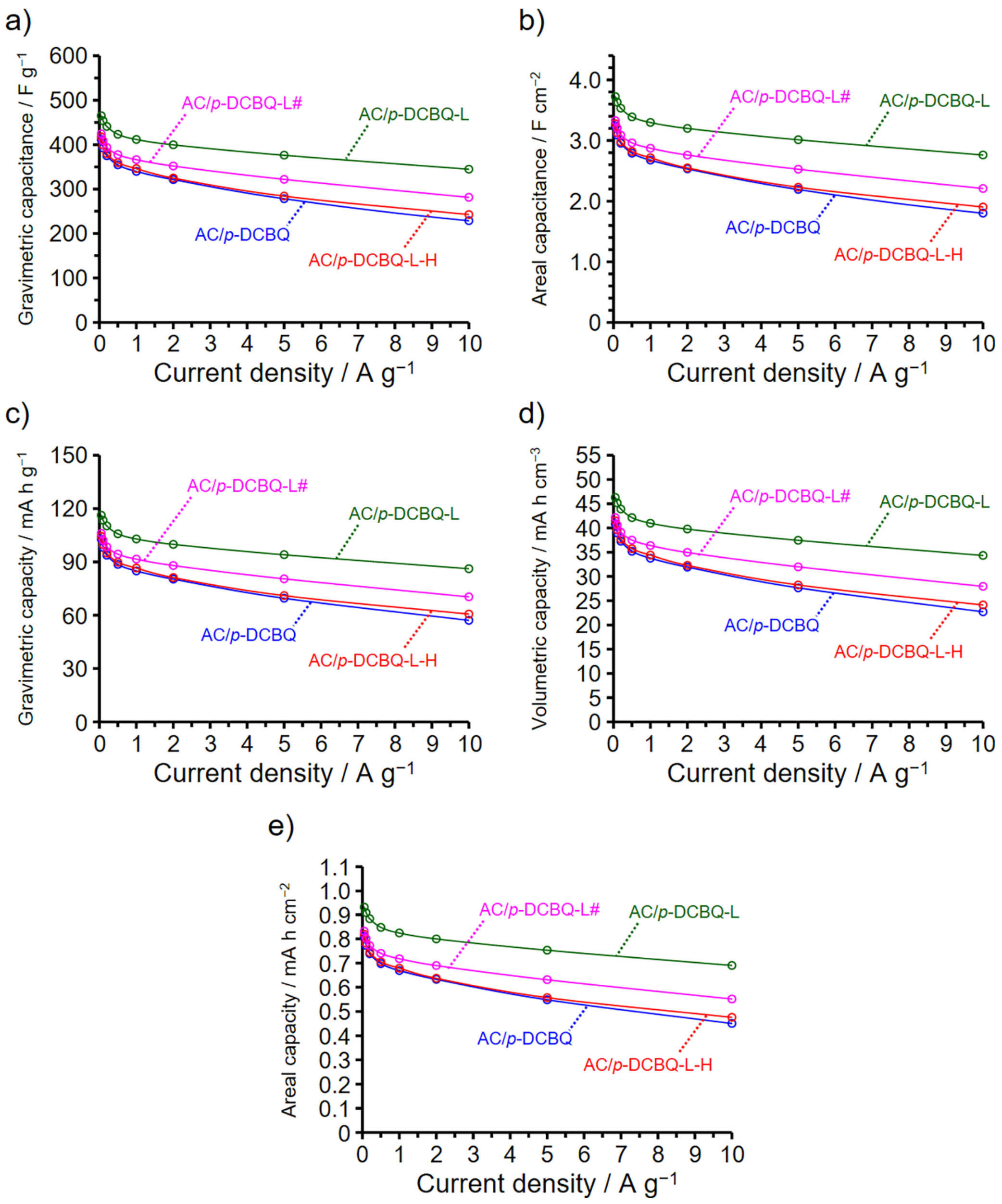

Fig. S17. Results of electrochemical measurements performed at 25 ºC using aqueous 1 M H_2SO_4 electrolyte and a three-electrode cell for AC/*p*-DCBQ-L, AC/*p*-DCBQ-L-H, and AC/*p*-DCBQ-L#. For comparison, the results of AC/*p*-DCBQ are shown together. (a and b) (a) Gravimetric and (b) areal capacitances. (c–e) (c) Gravimetric, (d) volumetric, and (e) areal capacities.

S13. Results of the electrochemical measurements for AC/*p*-DMBQ and AC/*p*-D^{t}BuBQ

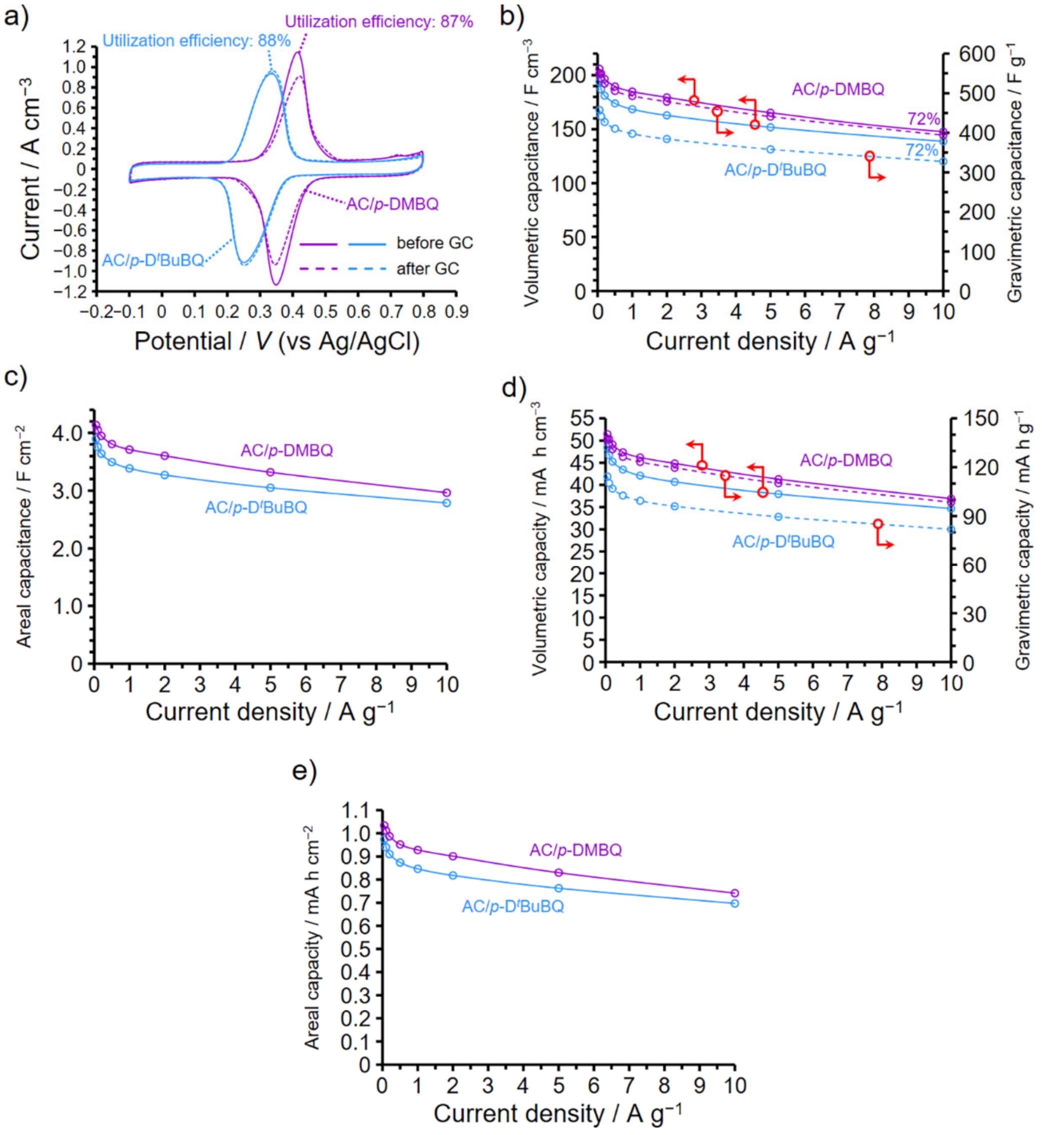

Fig. S18. Results of electrochemical measurements performed at 25 ºC using aqueous 1 M H_2SO_4 electrolyte and a three-electrode cell for AC/*p*-DMBQ and AC/*p*-D^{t}BuBQ. (a) Cyclic voltammograms collected at 1 mV s^{-1}. (b) Volumetric and gravimetric capacitances. (c) Areal capacitances. (d) Volumetric and gravimetric capacities. (e) Areal capacities.

S14. Results of the nitrogen adsorption/desorption measurement of AC/*p*-DCBQ-L, AC/*p*-DCBQ-L#, and AC/*p*-DCBQ-L-H

Fig. S19 shows the results of the nitrogen adsorption/desorption measurements for AC/*p*-DCBQ-L, AC/*p*-DCBQ-L#, and AC/*p*-DCBQ-L-H, shown alongside the result of AC/*p*-DCBQ for comparison. Their BET surface areas and pore volumes are summarized in Table S6 together with the normalized values. Although there is a slight difference in the pore size distribution (Fig. S19b), a significant difference is not observed in their isotherms (Fig. S19a), BET surface areas, and pore volumes.

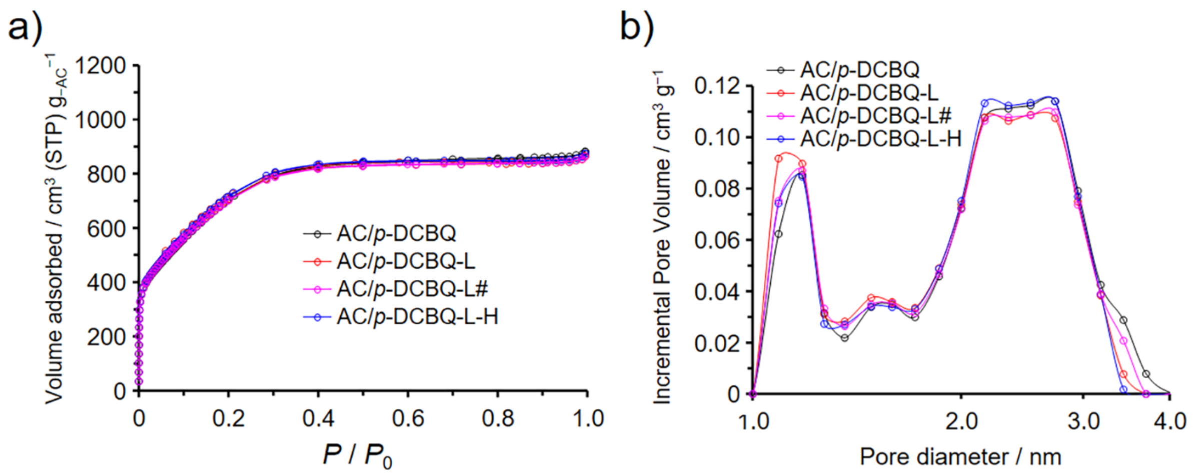

Fig. S19. (a) Nitrogen adsorption/desorption isotherms and (b) pore size distributions calculated by the DFT method based on the carbon slit pores (N_2, 77K) for AC/*p*-DCBQ-L, AC/*p*-DCBQ-L#, and AC/*p*-DCBQ-L-H, shown alongside the results of AC/*p*-DCBQ for comparison.

Table S6. BET surface areas and pore volumes of AC/*p*-DCBQ-L and AC/*p*-DCBQ-L-H.

Samples	wt%	S_{BET}[a)] [m^2 g^{-1}]	V_{total} [cm^3 g^{-1}]	V_{micro} [cm^3 g^{-1}]	V_{meso} [cm^3 g^{-1}]	S_{BET}[b)] [m^2 g^{-1}]	V_{total}[b)] [cm^3 g^{-1}]	V_{micro}[b)] [cm^3 g^{-1}]	V_{meso}[b)] [cm^3 g^{-1}]
AC/*p*-DCBQ-L	26.1	1960	0.96	0.60	0.36	2650	1.30	0.81	0.49
AC/*p*-DCBQ-L#	26.1	1940	0.96	0.59	0.37	2630	1.31	0.80	0.50
AC/*p*-DCBQ-L-H	26.1	1990	0.97	0.60	0.38	2700	1.32	0.81	0.51

[a)] BET surface area; [b)] Normalized values per 1 g of AC.

S15. Voltammograms of the 1st and 4th cycles for AC and AC/TMBQ

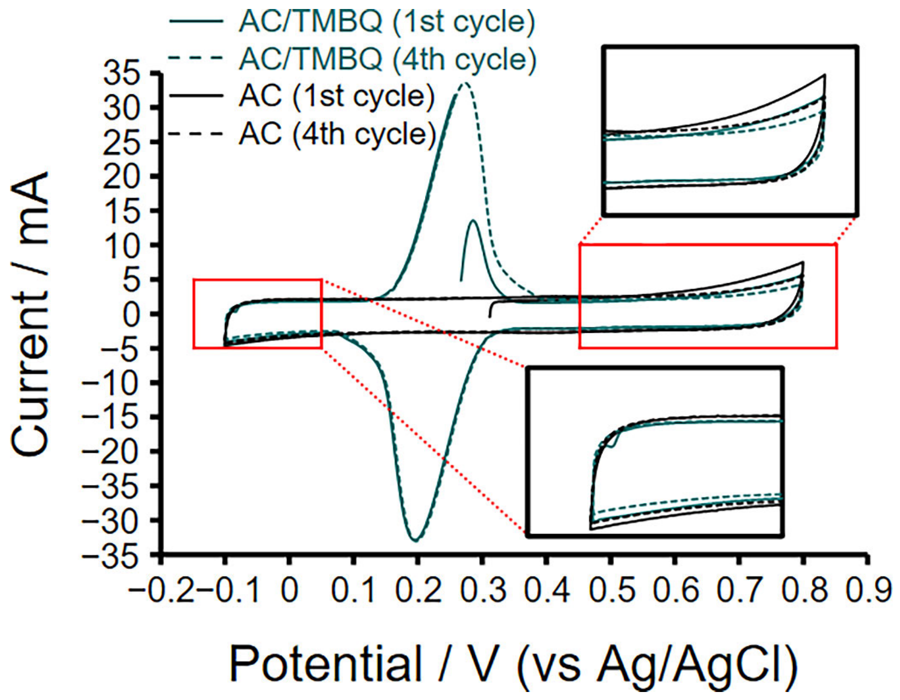

Fig. S20. Voltammograms of AC and AC/TMBQ at the first and fourth cycles measured at 1 mV s^{-1} using aqueous 1 M H_2SO_4 electrolyte and a three-electrode cell (−0.1~0.8 V, 25 ºC).

References

1. H. Itoi, Y. Yasue, K. Suda, S. Katoh, H. Hasegawa, S. Hayashi, M. Mitsuoka, H. Iwata and Y. Ohzawa, *ACS Sustainable Chem. Eng.*, 2017, **5**, 556-562.
2. H. Itoi, H. Hasegawa, H. Iwata and Y. Ohzawa, *Sustainable Energy Fuels*, 2018, **2**, 558-565.
3. H. Itoi, S. Maki, T. Ninomiya, H. Hasegawa, H. Matsufusa, S. Hayashi, H. Iwata and Y. Ohzawa, *Nanoscale*, 2018, **10**, 9760-9772.
4. H. Itoi, S. Tazawa, H. Hasegawa, Y. Tanabe, H. Iwata and Y. Ohzawa, *RSC Adv.*, 2019, **9**, 27602-27614.
5. H. Itoi, Y. Kasai, H. Hasegawa, K. Yamamoto, H. Iwata and Y. Ohzawa, *Chem. Phys. Lett.*, 2020, **755**, 137795.
6. H. Itoi, T. Ninomiya, H. Hasegawa, S. Maki, A. Sakakibara, R. Suzuki, Y. Kasai, H. Iwata, D. Matsumura, M. Ohwada, H. Nishihara and Y. Ohzawa, *J. Phys. Chem. C*, 2020, **124**, 15205-15215.
7. H. Itoi, S. Kotani, Y. Tanabe, Y. Kasai, R. Suzuki, M. Miyaji, H. Iwata and Y. Ohzawa, *Electrochim. Acta*, 2020, **362**, 137119.
8. K. P. O'Flynn and K. T. Stanton, *Cryst. Growth Des.*, 2012, **12**, 1218-1226.
9. A. Rochefort and J. D. Wuest, *Langmuir*, 2009, **25**, 210-215.
10. R. Bhowal, S. Biswas, A. Thumbarathil, A. L. Koner and D. Chopra, *J. Phys. Chem. C*, 2019, **123**, 9311-9322.
